# Tetraspanins are involved in *Burkholderia pseudomallei*-induced cell-to-cell fusion of phagocytic and non-phagocytic cells

**DOI:** 10.1038/s41598-020-74737-y

**Published:** 2020-10-21

**Authors:** Tanes Sangsri, Natnaree Saiprom, Alisa Tubsuwan, Peter Monk, Lynda J. Partridge, Narisara Chantratita

**Affiliations:** 1 Department of Microbiology and Immunology, Faculty of Tropical Medicine, Mahidol University, 420/6 Rajvithi Road, Bangkok, 10400 Thailand; 2 Institute of Molecular Biosciences, Mahidol University, Nakhon Pathom, 73170 Thailand; 3 Department of Infection, Immunity and Cardiovascular Disease, School of Medicine, University of Sheffield, Beech Hill Road, Sheffield, S10 2RX UK; 4 Department of Molecular Biology and Biotechnology, University of Sheffield, Western Bank, Sheffield, S10 2TN UK; 5 Mahidol-Oxford Tropical Medicine Research Unit, Faculty of Tropical Medicine, Mahidol University, Bangkok, 10400 Thailand

**Keywords:** Microbiology, Clinical microbiology, Infection

## Abstract

Tetraspanins are four-span transmembrane proteins of host cells that facilitate infections by many pathogens. *Burkholderia pseudomallei* is an intracellular bacterium and the causative agent of melioidosis, a severe disease in tropical regions. This study investigated the role of tetraspanins in *B. pseudomallei* infection. We used flow cytometry to determine tetraspanins CD9, CD63, and CD81 expression on A549 and J774A.1 cells. Their roles in *B. pseudomallei* infection were investigated in vitro using monoclonal antibodies (MAbs) and recombinant large extracellular loop (EC2) proteins to pretreat cells before infection. Knockout of CD9 and CD81 in cells was performed using CRISPR Cas9 to confirm the role of tetraspanins. Pretreatment of A549 cells with MAb against CD9 and CD9-EC2 significantly enhanced *B. pseudomallei* internalization, but MAb against CD81 and CD81-EC2 inhibited MNGC formation. Reduction of MNGC formation was consistently observed in J774.A1 cells pretreated with MAbs specific to CD9 and CD81 and with CD9-EC2 and CD81-EC2. Data from knockout experiments confirmed that CD9 enhanced bacterial internalization and that CD81 inhibited MNGC formation. Our data indicate that tetraspanins are host cellular factors that mediated internalization and membrane fusion during *B. pseudomallei* infection. Tetraspanins may be the potential therapeutic targets for melioidosis.

## Introduction

Tetraspanins are a superfamily of four-span transmembrane proteins distributed in multicellular organisms^1^. The proteins consist of four transmembrane domains, which can generate a protein structure containing one small extracellular loop (EC1) and one large extracellular loop (EC2). The function of tetraspanins depends on their ability to be laterally associated with the partner molecules, e.g., immunoglobulin superfamily members, integrins, and signalling molecules, to form functional assemblies as termed tetraspanin enriched microdomains (TEMs)^2,3^. Evidence is emerging and suggesting that TEMs are involved in many infectious diseases caused by viruses, bacteria, and protozoa and may act as gateways for infections^3,4^. TEMs may participate in various stages of infective processes, from facilitating binding to activating signalling pathways. Disruption of the TEM function may disorganize many potential receptors and interferes with the binding site of pathogens. Novel anti-infections targeting tetraspanins is a new strategy to inhibit the process of these infections.

*Burkholderia pseudomallei* is the causative agent of melioidosis, a fatal disease in tropical regions, endemic in Southeast Asia, and northern Australia^5^. The predicted burden of disease is about 165,000 cases, which includes 89,000 deaths^6^. The clinical manifestation of melioidosis ranges from acute to chronic infections with pneumonia and septicaemia being the most common presentations^7^. The mortality rate is 10 to 50% worldwide, and approximately 35% in Thailand. The death from melioidosis is often caused by delays in treatment or because of complications in clinical recognition and diagnosis^5,8,9^. Due to the high mortality rate, intrinsic antibiotic resistance, low infectious dose, aerosol route of infection, and no vaccine available, melioidosis is a public health concern in tropical countries. *B. pseudomallei* is an environmental bacterium, but it is classified as a CDC tier 1 select agent, a potential biothreat. It can infect humans and animals by inoculation, inhalation, and ingestion. *B. pseudomallei* can infect and survive within either phagocytic or non-phagocytic cells. After infection, the bacteria multiply in the cytoplasm and induce cell-to-cell fusion or multinucleated giant cell formation (MNGC). This process is crucial for spreading from infected cells to neighbouring cells. In this way, the bacteria can avoid exposure to host immune response or antibiotics^10^. Once the infection is established, *B. pseudomallei* may disseminate to many organs^5^. Although *B. pseudomallei* has been reported to use several virulence factors for invasion, the potential host molecules that contribute to bacterial and host interactions are poorly understood.

*B. thailandensis* is a closely-related species of *B. pseudomallei. B. thailandensis* can infect both phagocytic and non-phagocytic cells and subsequently induce MNGC formation in vitro similar to *B. pseudomallei*^10,11^. Tetraspanins have been widely implicated in the regulation of various cell: cell fusion processes, including viral syncytium formation^12–15^. We have also recently demonstrated that reagents targeting tetraspanins CD9, CD63, and CD81 could affect *B. thailandensis*-induced MNGC in mouse macrophage cell lines. In brief, recombinant proteins corresponding to the EC2s of these tetraspanins inhibited MNGC formation while antibodies to CD9 and CD81 enhanced MNGC formation, and a macrophage cell line generated from a CD9KO mouse showed enhanced MNGC formation^16^. This led us to hypothesize that tetraspanins might play a role in *B. pseudomallei* infection and bacterial spreading between host cells during melioidosis.

In order to investigate the role of tetraspanins in *B. pseudomallei* infection, we used monoclonal antibodies (MAbs) specific to the large extracellular EC2 domain of tetraspanins and recombinant EC2 proteins of CD9, CD63, and CD81 to pretreat a human epithelial cell line A549 and a mouse macrophage cell line J774A.1 before infection. Mouse macrophage cell lines have been widely used as the in vitro model for *B. pseudomallei* and *B. thailandensis* infection and MNGC formation^16–21^. The mouse tetraspanin CD9 is about 90% homology to Homo sapiens CD9 and has been used to represent mammalian cells in sperm-egg fusion assay^22,23^. We determined the expression of tetraspanins on host cells by flow cytometry and assessed the number of bacterial adhesion and internalization to these cells by colony count. MNGC formation and MNGC size of infected cells was determined by imaging analyses using light microscopy and confocal microscopy. We also performed tetraspanin genes knock out on cells using the CRISPR/Cas9 system and confirmed a role for host tetraspanins for *B. pseudomallei* infection. Finally, the role of tetraspanins for infections was compared between *B. pseudomallei* and *B. thailandensis*.

## Results

### Expression of tetraspanins on uninfected A549 and J774A.1 cells

The role of tetraspanins in *B. pseudomallei* infection and MNGC formation were investigated in two cell lines. A549 represented a non-phagocytic human epithelial cell, and J774A.1 represented a phagocytic mouse macrophage cell. To determine if tetraspanins are expressed on the cell surface of the cells, we used flow cytometry and antibodies that recognise CD9, CD63, and CD81. We observed in both A549 and J774A.1 that the MFI level of cells treated with antibodies against CD9 and CD81 were significantly higher than those treated with isotype controls (Supplementary Fig. S1). In contrast, the MFI level of cells treated with the antibody against CD63 was not different from isotype controls. These results indicated that CD9 and CD81 expressed on A549 and J774A.1 at a relatively high level but that CD63 was poorly expressed on the cell surface.

### Expression of tetraspanins on *B. pseudomallei* infected A549 and J774A.1 cells

We next investigated the level of these tetraspanins expression on A549 and J774A.1 cells during *B. pseudomallei* infection. We used flow cytometry and specific antibodies to determine CD9, CD63, and CD81 expressions on cells at 1, 4, and 12 h after infection at MOI 100 or 30. The result showed that expressions of CD9, CD63, and CD81 on both cells were not significantly different between *B. pseudomallei* infected cells and non-infected cells at all-time points (Supplementary Fig. S2). These results demonstrate that *B. pseudomallei* infections did not alter CD9, CD63, and CD81 expression on these cells.

### Effect of anti-tetraspanin MAbs and recombinant EC2 proteins on *B. pseudomallei* adhesion and internalization

**Figure 1:**
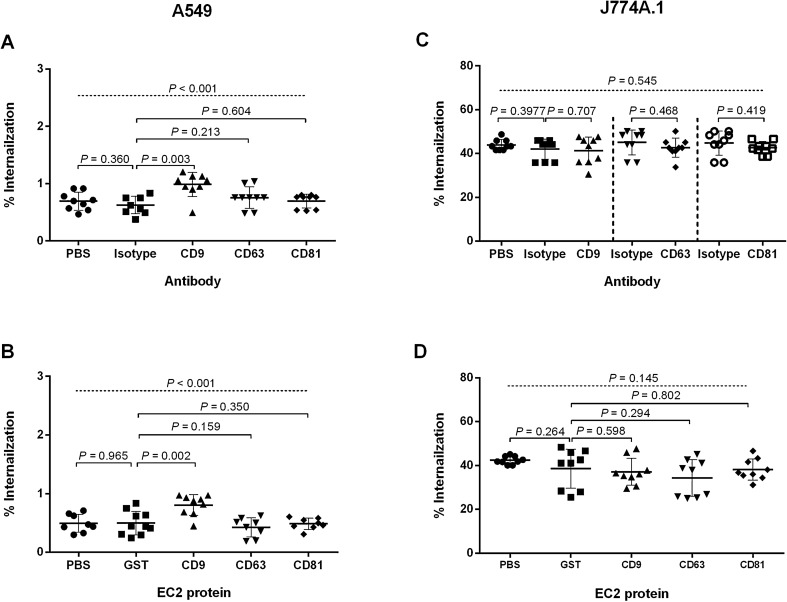
Effect of anti-tetraspanin MAbs and recombinant EC2 proteins pretreatment on *B*. *pseudomallei* K96243 internalisation to A549 and J774A.1 cells. The internalisation of *B. pseudomallei* K96243 to A549 cells (**A**,**B**) and J774A.1 (**C**,**D**) were performed at MOI of 100 and 30. A and C show the effect of anti-tetraspanin MAbs. (**B**,**D**) show the effect of recombinant EC2 protein pretreatment. Data represent individual scatter plots and the mean ± standard deviation from three independent experiments; each experiment was performed in triplicate. Dashed lines represent *P* values of ANOVA test, and solid lines represent *P* value of *t* tests. The graphs were created using GraphPad Prism software version 6.0 (GraphPad Software Inc, La Jolla, CA).

To determine the role of tetraspanins in *B. pseudomallei* adhesion and internalization in A549 and J774A.1 cells, we used MAbs specific to tetraspanins as well as recombinant EC2 proteins to treat cells for 1 h before infection. The controls were cells incubated with PBS or IgG isotype-matched MAbs or GST. The result showed no difference in bacterial adherence between cells pretreated with these reagents and isotype controls or PBS in both cell types (Supplementary Fig. S3). Since J774A.1 cells are capable of phagocytosis, we considered that the measurement of associated bacteria at this point might be both bacterial adhesion and internalization. We, therefore, investigated the role of tetraspanin in adhesion in J774A.1 after blocking phagocytosis with 2 µg/ml cytochalasin D^17^. The result also showed no significant difference in adherence between J774A.1 cells pretreated with anti-tetraspanin MAbs and isotype controls or PBS, which was similar to the result of the experiment without cytochalasin D. The data suggest that tetraspanins are not involved in bacterial adhesion in J774A.1 cells (Supplementary Fig. S4). For internalisation, only pre-treatment with anti-CD9 MAb and CD9-EC2 significantly increased the percentage of bacterial internalisation into A549 cells (*P* = 0.003 for anti-CD9 MAb; *P* = 0.002 for CD9-EC2) (Fig. 1A,B). In contrast, none of these MAbs or EC2 proteins inhibited *B. pseudomallei* internalisation in J774.A1 cells. The data suggest different roles of CD9 for bacterial uptake between A549 and J774.1 cells.

### Effect of anti-tetraspanin MAbs and recombinant EC2 proteins on *B. pseudomallei*-induced MNGC formation

**Figure 2:**
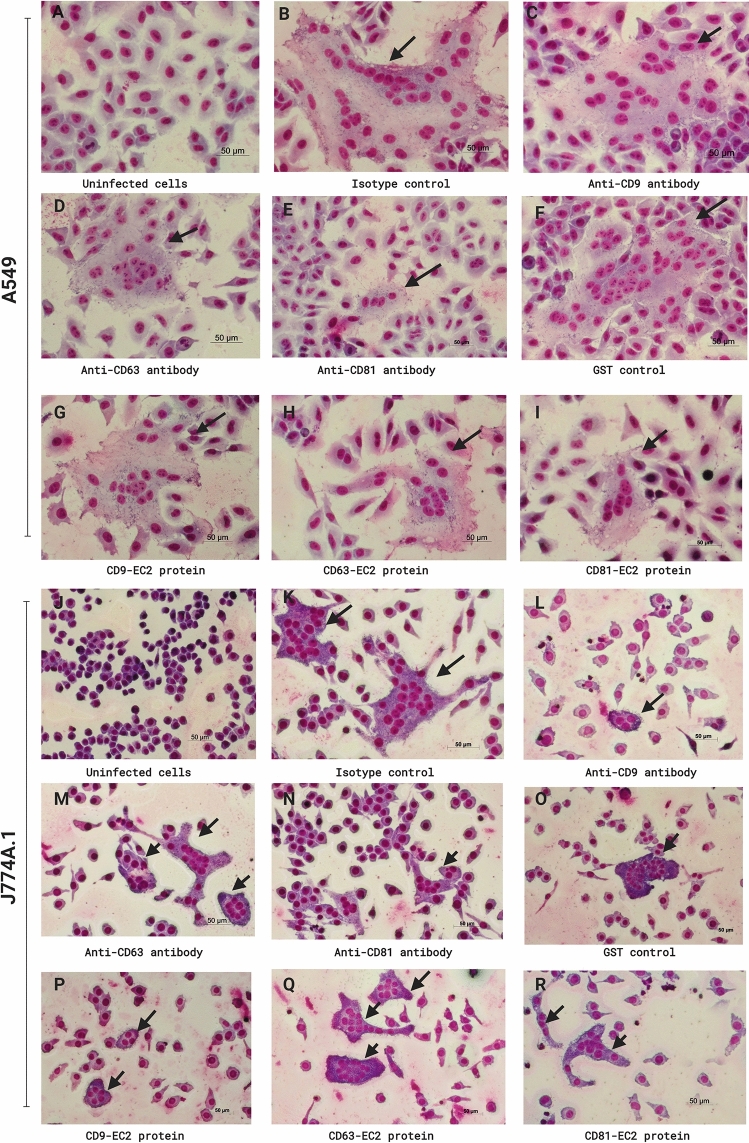
Giemsa-stained A549 cells (**A**–**I**) and J774A.1 cells (**J**–**R**) following *B. pseudomallei* infection. Cells were pretreated with anti-tetraspanin MAbs or isotype controls before infection. Cells were fixed, stained, and observed for MNGC formation at 12 h post-infection using a 20 × objective lens. Arrow indicates MNGC. The images were combined using BioRender.com (https://app.biorender.com).

**Figure 3:**
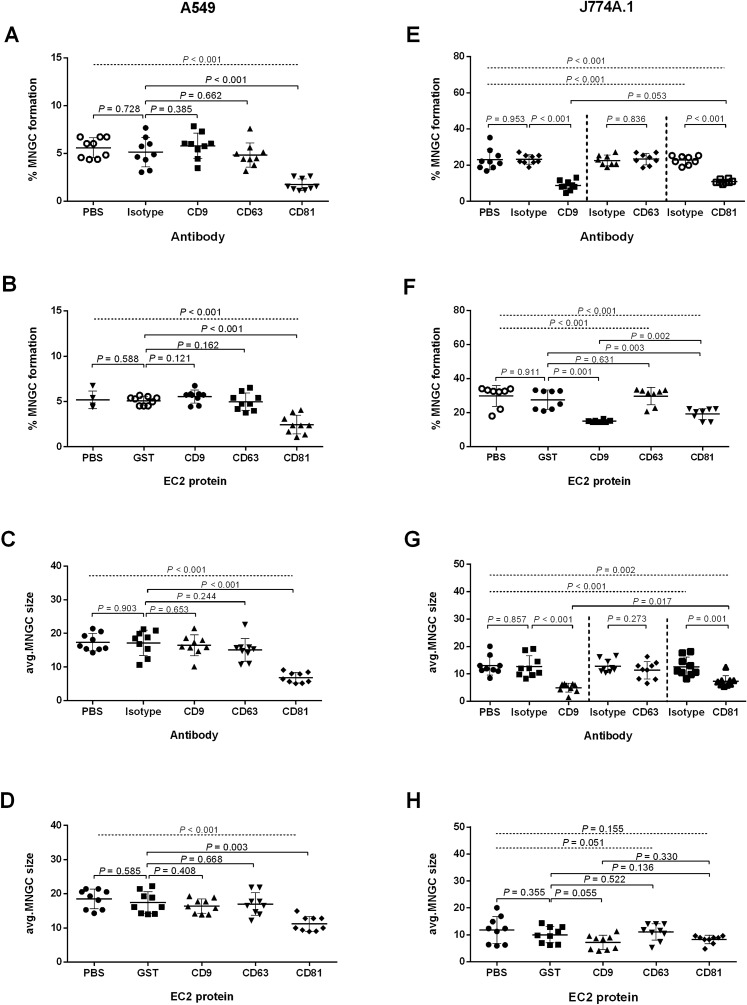
MNGC formation of A549 and J774A.1 cells induced by *B. pseudomallei* K96243 infection. A549 and J774A.1 cells were infected with *B. pseudomallei* at MOI of 100 and 30. Effect of anti-tetraspanin MAbs (**A**) and recombinant EC2 proteins (**B**) pretreatment on *B*. *pseudomallei*-induced MNGC formation of A549 cells. Effect of anti-tetraspanin MAbs (**C**) and recombinant EC2 proteins (**D**) pretreatment on average MNGC size of A549 cells. Effect of anti-tetraspanin MAbs (**E**) and recombinant EC2 proteins (**F**) pretreatment on *B*. *pseudomallei*-induced MNGC formation to J774A.1 cells. Effect of anti-tetraspanin MAbs (**G**) and recombinant EC2 proteins (**H**) pretreatment on average MNGC size of J774A.1 cells. Data represent individual scatter plots and the mean ± standard deviation from three independent experiments; each experiment was performed in triplicate. Dashed lines represent *P* values of ANOVA test, and solid lines represent *P* value of *t* tests. The graphs were created using GraphPad Prism software version 6.0 (GraphPad Software Inc, La Jolla, CA).

Anti-tetraspanin MAbs and recombinant EC2 proteins were used to investigate the role of tetraspanins during *B. pseudomallei*-induced MNGC formation in A549 and J774A.1 at 12 h post-infection. In A549 cells, the number of MNGC formation and MNGC size of cells treated with MAbs against CD9 and CD63 and their EC2 proteins were not different from those treated with isotype or GST controls (Figs. 2, 3A,B). Interestingly, treatment with MAb specific to CD81 and CD81-EC2 significantly inhibited the number of *B. pseudomallei*-induced MNGC formation by 68.3% and 53.8% (*P* < 0.001 for both comparisons), respectively (Figs. 2, 3A,B) and reduced in MNGC size by 60.1% (*P* < 0.001) and 41.1% (*P* = 0.003), respectively compared with cells treated with isotype-matched controls or GST control (Figs. 2, 3C,D).

In contrast, pretreatment of J774A.1 cells with MAbs against both CD9 and CD81 significantly inhibited the number of MNGC formation by 61.9% and 53.0% (*P* < 0.001 for both comparisons) (Figs. 2 and 3E) and reduced in MNGC size by 60.8% and 42.4% (*P* = 0.001), respectively when compared with cells treated with isotype controls (Figs. 2 and 3G). Likewise, cells treated with CD9-EC2 and CD81-EC2 significantly inhibited *B. pseudomallei*-induced MNGC formation by 42.4% (*P* = 0.001) and 31.5% (*P* = 0.003) compared with GST control (Figs. 2 and 3F). However, cells treated with CD9-EC2 and CD81-EC2 showed a reduction in MNGC size when compared with GST control (Fig. 2), but the results were not statistical significance (Fig. 3H).

To investigate whether the inhibition of *B. pseudomallei*-induced MNGC formation by CD9-EC2 and CD81-EC2 may increase at higher EC2 concentrations, pretreatments of J774A.1 were performed with CD9-EC2 and CD81-EC2 at 10, 20, 50, and 100 µg/ml. We did not observe different MNGC formation between cells treated with PBS or GST control and those pretreated with ten µg/ml CD9-EC2, but there was a decrease in MNGC formation when cells were treated with 20, 50, and 100 µg/ml of CD9-EC2 concentrations (Supplementary Fig. S5A). We further tested the effect of combined CD9-EC2 and CD81-EC2 pretreatment in J774A.1 cells, but there was no increase in inhibitory effect from the results of pretreatment with CD9-EC2 alone or with CD81-EC2 alone (Supplementary Fig. S5B).

We next confirmed the role tetraspanins in *B. pseudomallei*-induced MNGC formation by treating the cells with anti-tetraspanin MAbs at 5 h post-infection, the time in which after bacterial internalization and before MNGC formation took place. Nevertheless, we observed no different results from those of adding anti-MAbs before infection (Supplementary Fig. S6). Furthermore, Giemsa staining of MNGC revealed that *B. pseudomallei* were able to multiply in the cytosol of A549 cells pretreated with anti-CD81 antibody or J774A.1 cells pretreated with anti-CD9 antibody similar to those cells pretreated with isotype-matched controls (Supplementary Fig. S7). Altogether, our data confirmed that CD9 and CD81 tetraspanins are involved in cell fusion induced by *B. pseudomallei*.

### The localisation of tetraspanin during *B. pseudomallei*-induced MNGC formation

**Figure 4:**
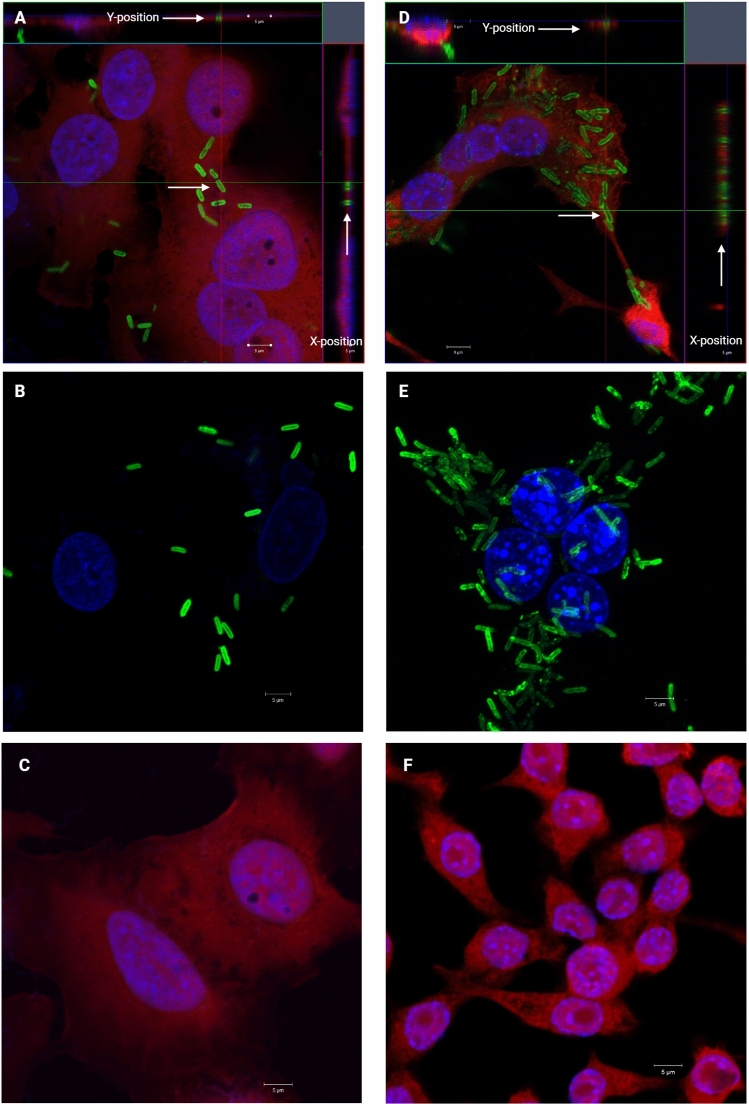
Confocal fluorescence microscopy z-stack imaging of A549 cells infected with *B. pseudomallei* K96243 at MOI 25 (**A**) and J774A.1 cells infected with *B. pseudomallei* K96243 at MOI 10 (**D**) at 8 h. Z-stack images were collected at 4.0-µm sections, and the red and green lines indicate the orthogonal planes of the *y–z* and *x–z* projections, respectively. Bacteria were stained with anti-*B. pseudomallei* capsular polysaccharide antibody-conjugated Alexa Fluor 488 (green). (**B**) Shows infected A549 cells stained with an Alexa Fluor 555 conjugated IgG1 isotype control of mouse anti-human CD81. (**E**) Shows infected J774A.1 cells stained with an Alexa Fluor 555 conjugated IgG2b isotype control of rat anti-mouse CD9. C and F show uninfected A549 and J774A.1 cells stained with an Alexa Fluor 555 conjugated mouse anti-human CD81 or rat anti-mouse CD9 (red) and nuclei were stained with Hoechst 33,258 (blue). Arrows indicate the position of bacteria and tetraspanins during *B. pseudomallei*-induced cell-to-cell fusion. Bars, 5 µm. Images were visualised using a confocal microscope (LSM 700; Carl Zeiss), a 100 × objective. The images were combined using BioRender.com (https://app.biorender.com).

Since we observed that CD9 and CD81 were involved with MNGC formation induced by *B. pseudomallei* in J774A.1 and CD81 significantly involved in this process in A549 cells, we determined (1) CD9 and CD81 localisation in J774A.1 and (2) CD81 localisation in A549 cells during *B. pseudomallei*-induced MNGC formation by immunofluorescence staining and confocal microscopy. CD9 and CD81 were examined using specific antibodies and LSM, Z-stack analysis. In uninfected cells, immunofluorescence staining showed that CD9 and CD81 distributed in all areas of the cells (Fig. 4 and Supplementary Fig. S8). J774A.1 and A549 cells were infected with *B*. *pseudomallei* K96243. Our investigations revealed the presence of bacteria within the cytoplasm of the infected J774A.1 and A549 cells (Fig. 4). CD81 was observed in all areas of infected A549 cells (Fig. 4A), and CD9 was distributed in infected J774A1 cells (Fig. 4D). Cells treated with isotype-matched controls were negative for CD81 and CD9 (Fig. 4B,E, respectively). The results also demonstrated that tetraspanins CD9 and CD81 were located with *B. pseudomallei* at membrane protrusion ends.

### Generation of tetraspanins gene knockout in A549 cells

**Figure 5:**
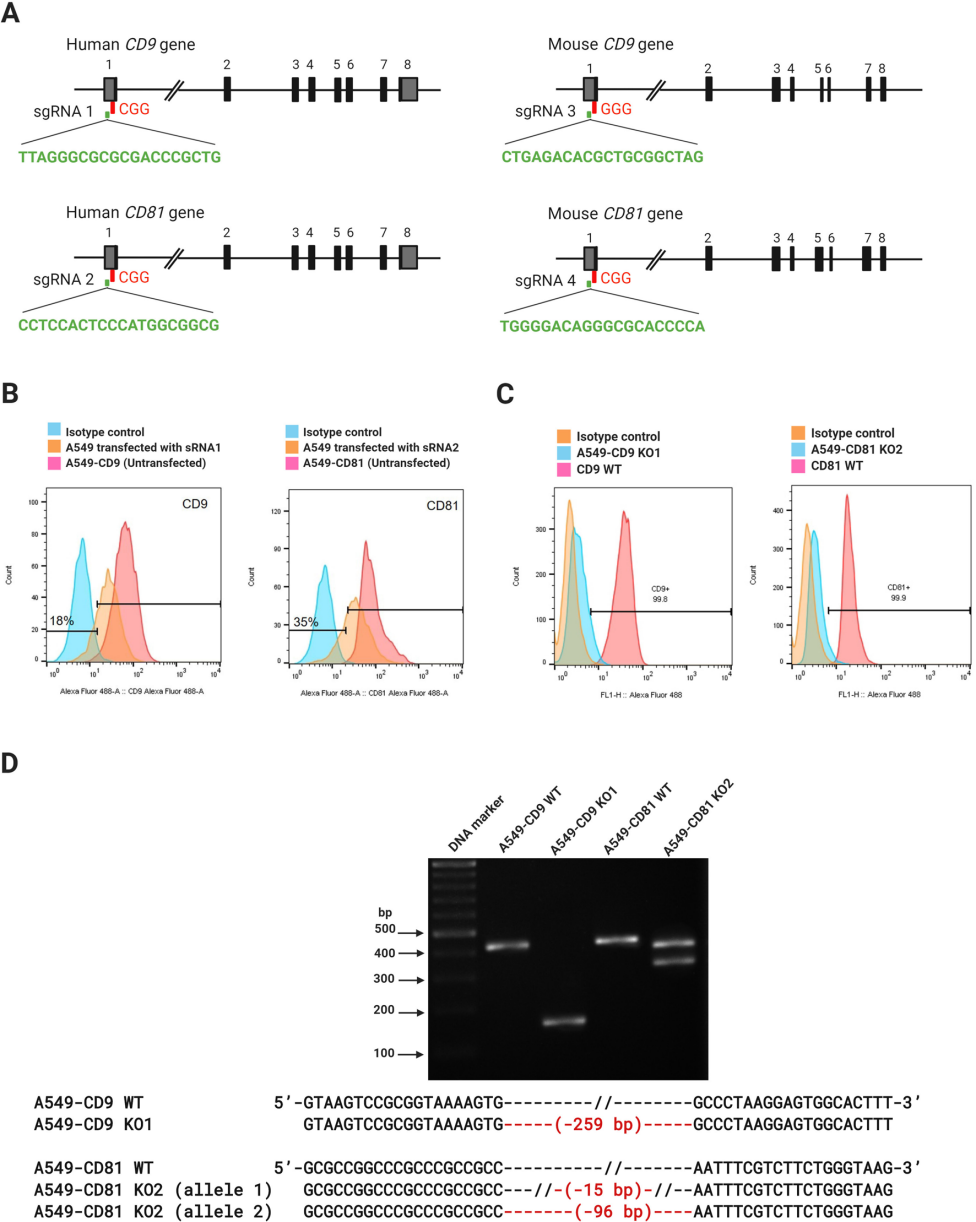
Detection and confirmation of CD9 and CD81 genes knockout by CRISPR/cas9 system. The gRNA sequences were consisting of a 20-nucleotides pair with the DNA target, directly upstream of a requisite 5′-NGG adjacent motif (PAM; red) (**A**). The transfection efficiency of CD9 targeting gRNA1 and CD81 targeting gRNA2 in A549 cells was analysed by flow cytometry (**B**). The expression of CD9 and CD81 on the cell surface was analysed by flow cytometry, as shown in histogram (**C**). DNA cleavage by CRISPR/cas9 was detected by PCR and confirmed by sequencing (**D**). The deletion regions are shown in red. The images were combined using BioRender.com (https://app.biorender.com).

To confirm the role of tetraspanins, CD9, and CD81 in *B. pseudomallei* internalization and MNGC formation in A549 and J774A.1 cells, we generated CD9 and CD81 gene knock out cell lines using the CRISPR/Cas9 system. sgRNA sequences targeting both human and mouse CD9 and CD81 were designed (Fig. 5A) and cloned into the PX459V2.0. A549 or J774A cells were transfected with individual plasmids expressing Cas9 and the corresponding sgRNA. As assessed by flow cytometry, gRNA1 and gRNA2 were able to disrupt CD9 and CD81 expression in A549 cells with efficiencies of 18% and 35%, respectively (Fig. 5B). Transfection of J774A.1 with the plasmids resulted in massive cell death, and the number of living cells was not enough for further analysis.

Only CD9 and CD81 knockout A549 cells were further sorted by FACS. The sorted cells were isolated into a single cell by limiting dilution. Two single clones, A549-CD9KO1 and A549-CD81KO2, were expanded and selected for further analysis. Flow cytometry analysis confirmed the absence of CD9 and CD81 expression on the cell surface of those cells when compared with the unedited control (Fig. 5C). To characterize the nature of the mutation in these cells, PCR amplicon spanning the Cas9-sgRNA cleavage site was amplified from the two individual clones and analyzed the products on an agarose gel. Only one PCR fragment was amplified in the A549-CD9KO1 clone, while two PCR fragments were detected in the A549-CD81KO2 clone. The products from both A549-CD9KO1 and A549-CD81KO2 clones were smaller than the unedited control DNA, indicating that gene deletions were introduced into the genomic sequence of those cells (Fig. 5D and Supplementary Fig. S9). To confirm these findings, individual PCR fragments were purified from the gel and subjected to direct sequencing. Sequence from A549-CD9KO1 displayed a 259-bp deletion in the 5′ untranslated region of CD9 in both alleles (Fig. 5D). This would inhibit CD9 expression via translation control. Two deleted alleles were identified from A549-CD81-KO2 (Fig. 5D). One exhibited a 15-bp deletion in the 5′ untranslated region of CD81 gene, which would affect its expression. Another allele revealed a 96-bp deletion that generated truncated protein that lacked the intracellular N terminal domain and EC1. The lack of EC1 would destabilize EC2 folding and further disrupt EC2 function^24^. The lack of the protein expressions on the cell surface was confirmed by FACs analysis, Western blotting (Supplementary Fig. S10), and sequencing analysis, which indicated successful CD9 and CD81 gene knock out in A549-CD9KO1 and A549-CD81KO2 cell lines using the CRISPR/Cas9 system.

### Effect of tetraspanins gene knockout on *B. pseudomallei* internalisation and MNGC formation

**Figure 6:**
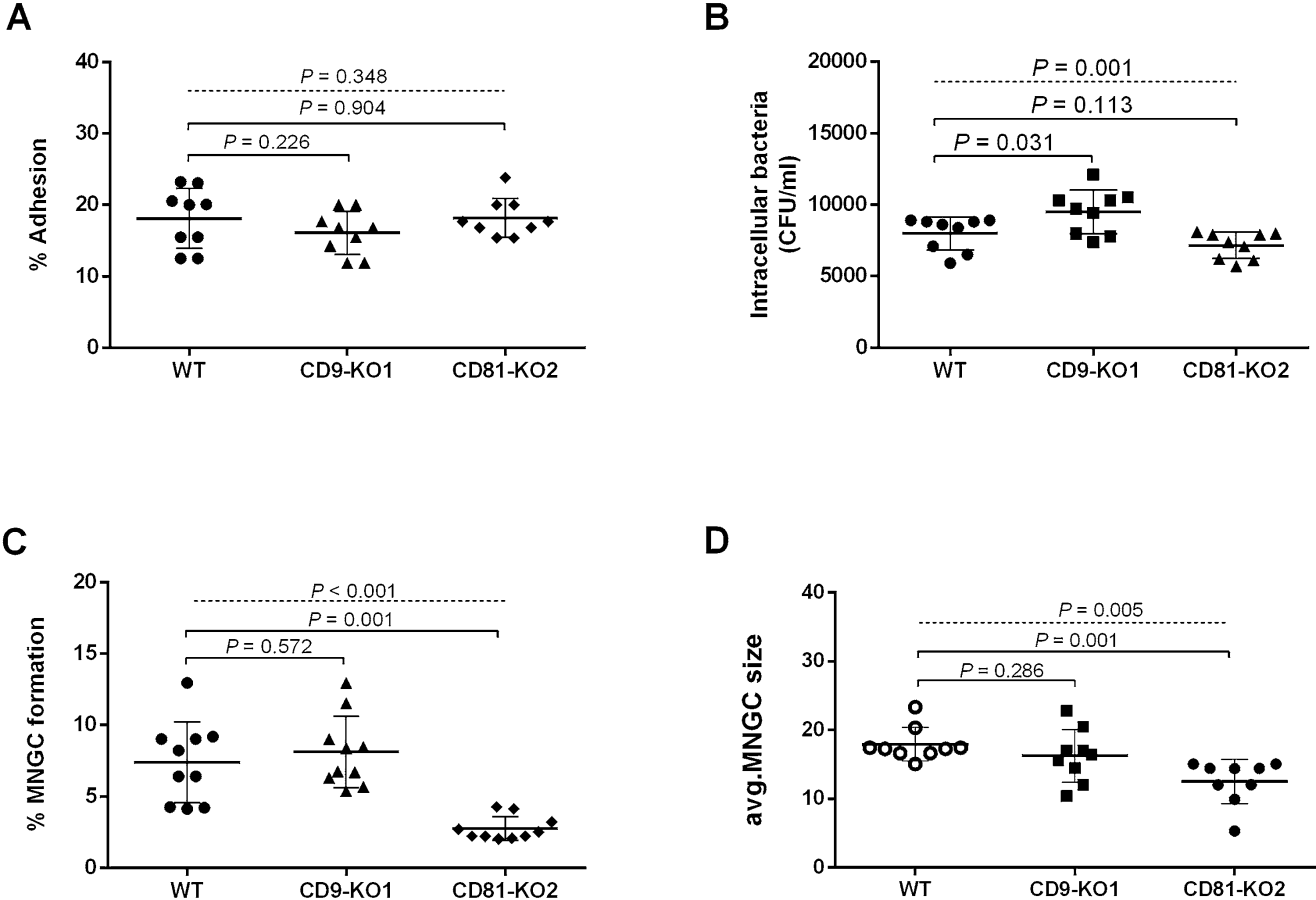
The effects of CD9 and CD81 genes knockout of A549 cells on *B. pseudomallei* infection. A549 cells were infected with *B. pseudomallei* at MOI 100 for 1, 4, and 12 h at 37 °C. Effect of gene knockout on *B. pseudomallei* adhesion (**A**)*,* internalization (**B**), MNGC formation (**C**), and MNGC size (**D**). Data represent the mean ± standard deviation Data represent individual scatter plots, and the mean ± standard deviation from three independent experiments, each experiment was performed in triplicate. Dashed lines represent *P* values of ANOVA test, and solid lines represent *P* value of *t* tests. The graph were created using GraphPad Prism software version 6.0 (GraphPad Software Inc, La Jolla, CA).

**Figure 7:**
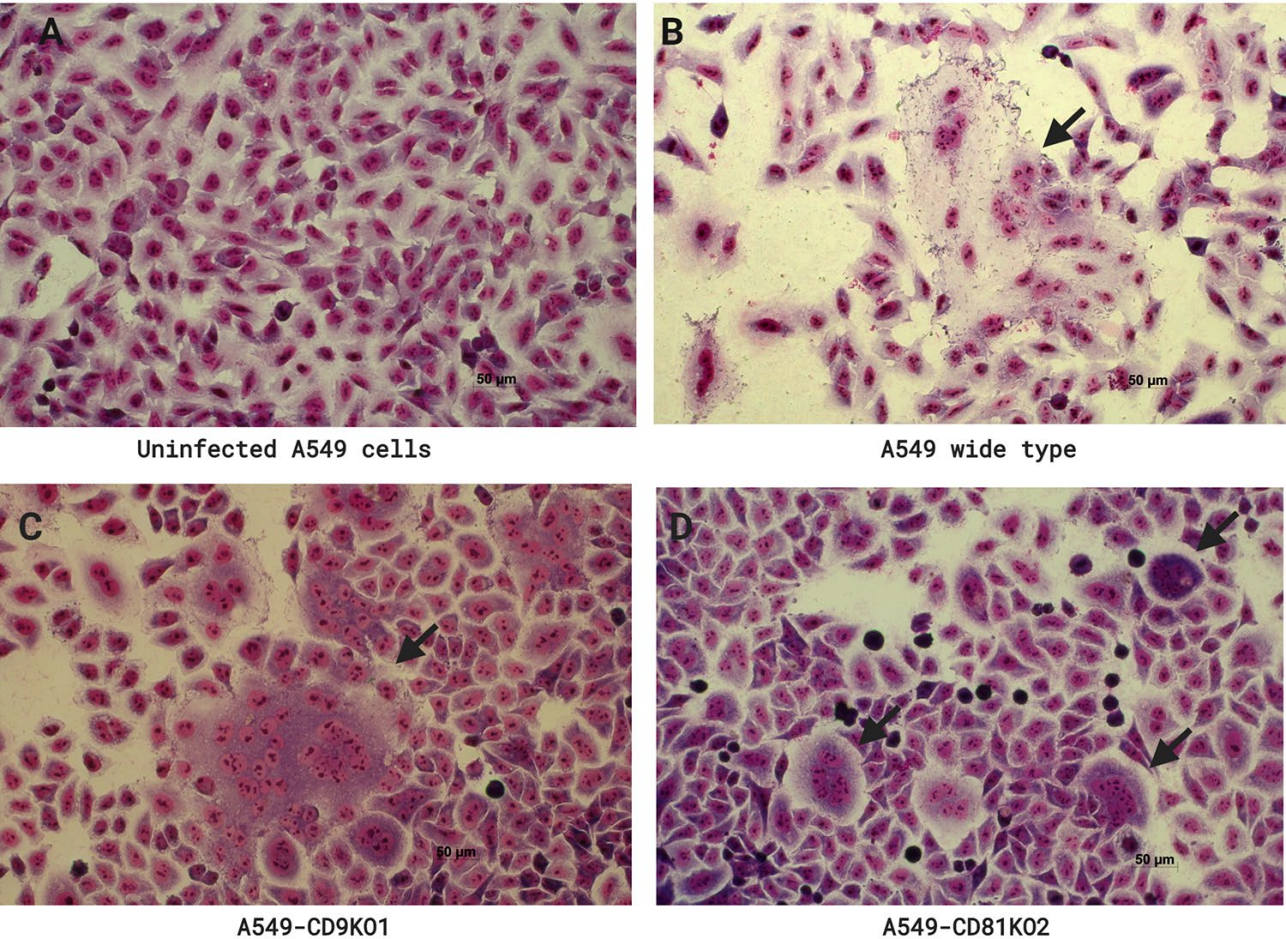
Giemsa-stained A549 cells wild type, A549-CD9KO1, and A549-CD81KO2 following *B. pseudomallei* K96243 infection. Cells were fixed, stained, and observed for MNGC formation at 12 h post-infection using a 20 × objective lens. Arrows indicate MNGC. The images were combined using BioRender.com (https://app.biorender.com).

The absence of CD9 and CD81 on the A549 cell surface did not show any effect on *B. pseudomallei* adhesion (Fig. 6A). As expected, infection of A549-CD9KO1 cells showed a significant increase in the number of *B. pseudomallei* internalisation compared with A549 WT cells (Fig. 6B). Moreover, image analyses by light microscopy demonstrated that A549-CD81KO2 showed significantly decreased *B. pseudomallei*-induced MNGC formation and a reduction of MNGC size (Figs. 6C,D, and 7, respectively). The result of the gene knockout experiment on A549 cells was consistent with those of pretreatment with anti-CD9 MAb and anti-CD81 MAb or recombinant EC2 proteins (Figs. 1, 2, 3). Taken together, these results suggest that tetraspanins CD9 and CD81 of host cells play a crucial role in *B. pseudomallei* internalisation and MNGC formation.

### Comparison of the effect of anti-tetraspanin MAbs on MNGC formation induced by *B. thailandensis* and *B. pseudomallei*

**Figure 8:**
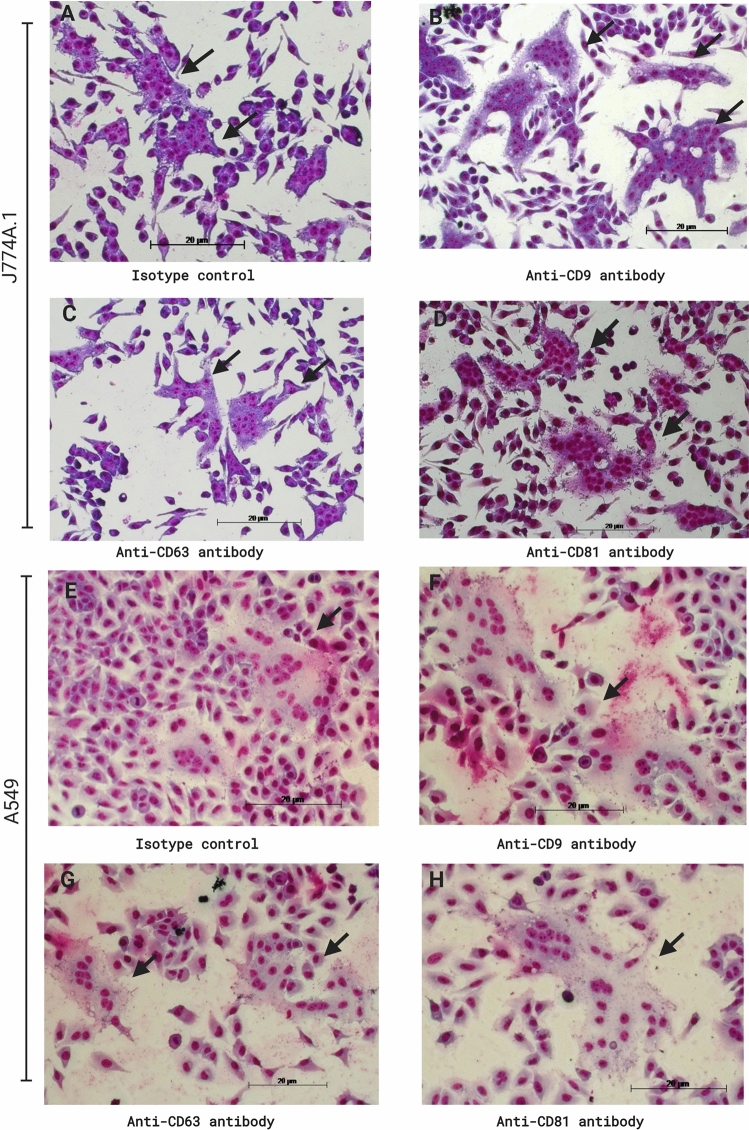
Giemsa-stained J774A.1 cells (**A**–**D**) and A549 cells (**E**–**H**) following *B. thailandensis* E264 infection. Cells were pretreated with anti-tetraspanin MAbs or isotype controls before infection. Cells were fixed, stained, and observed for MNGC formation at 12 h post-infection using a 20 × objective lens. Arrows indicate MNGC. The images were combined using BioRender.com (https://app.biorender.com).

**Figure 9:**
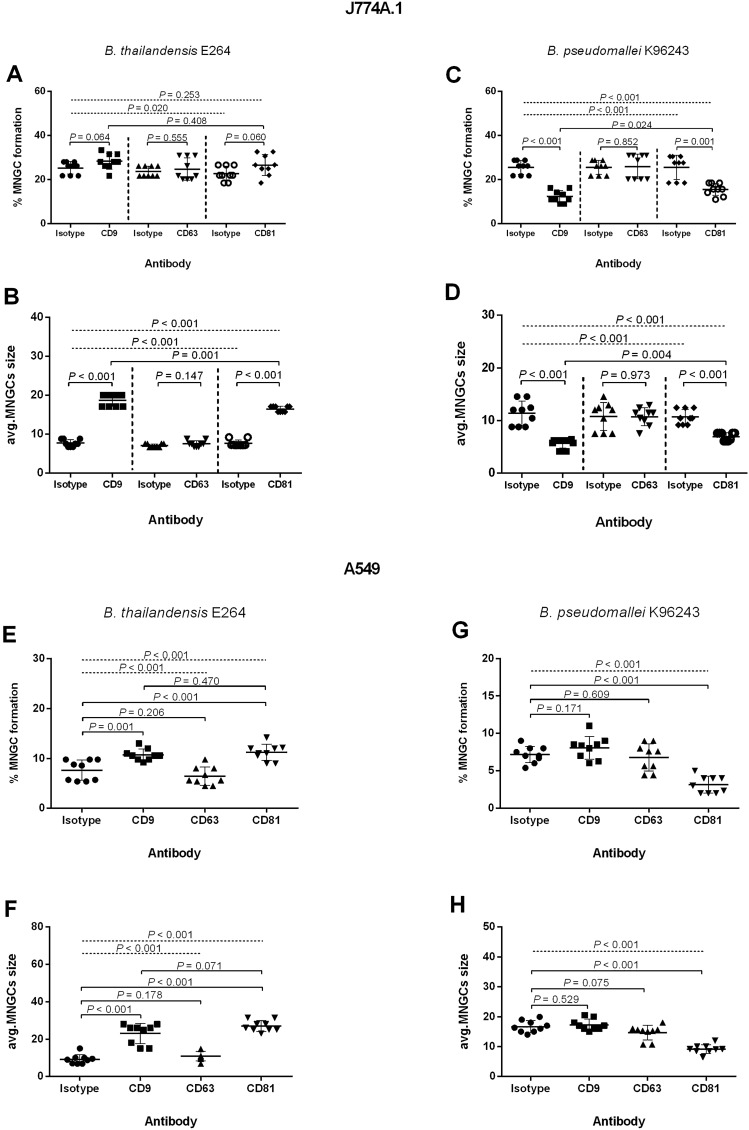
Effect of anti-tetraspanin MAbs on MNGC formation induced by *B. thailandensis* E264 and *B. pseudomallei* K96243 in J774A.1 and A549 cells. (**A**,**C**,**E**,**G**) show the effect of anti-tetraspanin MAbs pretreatment on MNGC formation. (**B**,**D**,**F**,**H**) show the effect of anti-tetraspanin MAbs pretreatment on average MNGC size. J774A.1 cells were infected with *B. thailendensis* (**A**,**B**) or *B. pseudomallei* (**C**,**D**) at MOI 30. A549 cells were infected with *B. thailandensis* (**E**,**F**) or *B. pseudomallei* (**G**,**H**) at MOI 100. Data represent individual scatter plots and the mean ± standard deviation from three independent experiments; each experiment was performed in triplicate. Dashed lines represent *P* values of ANOVA test, and solid lines represent *P* value of *t* tests. The graphs were created using GraphPad Prism software version 6.0 (GraphPad Software Inc, La Jolla, CA).

The inhibition of *B. pseudomallei-*induced MNGC formation by CD9 and CD81 GST-EC2 proteins is consistent with their effect on this process in *B. thailandensis* infected mouse macrophages^16^. However, our previous report demonstrated that in this system, anti-CD9 and anti-CD81 MAbs enhanced MNGC formation, while here, the same antibodies inhibited *B. pseudomallei-*induced MNGC formation (Figs. 2 and 3). We, therefore, compared the effect of the anti-tetraspanin MAbs on MNGC formation in J774A.1 and A549 cells infected with *B. thailandensis* and *B. pseudomallei* K96243. Indeed, the results showed different effects of anti-CD9 and anti-CD81 MAbs between cells infected with the two *Burkholderia* species. We observed that pretreatment with anti-CD9 and anti-CD81 MAbs enhanced *B. thailandensis*-induced MNGC formation and MNGC size in J774A.1 cells (Figs. 8, 9A,B), consistent with our previous work^16^. The results of *B. pseudomallei* infection were the same as in previous experiments (Figs. 2 and 3), demonstrating that in this system, anti-CD9 and anti-CD81 MAbs significantly inhibited MNGC formation and reduced MNGC size in J774A.1 cells (Fig. 9C,D).

In infected A549 cells, pretreatment with anti-CD9 and anti-CD81 MAbs also showed different effects on MNGC formation between *B. thailandensis* and *B. pseudomallei*. The results revealed pretreatment with anti-CD9 and anti-CD81 MAbs enhanced *B. thailandensis*-induced MNGC formation cells (Figs. 8 and 9E) and MNGC size (Fig. 9F), while pretreatment with anti-CD81 MAb significantly inhibited *B. pseudomallei*-induced MNGC formation (Fig. 9G) and decreased MNGC size (Fig. 9H).

## Discussion

Here, we demonstrated a role for host tetraspanins in *B. pseudomallei* internalisation and MNGC formation during *B. pseudomallei* infection in A549 and J774A.1 cell lines. In A549 lung epithelial cells, our results revealed that CD9 facilitated *B. pseudomallei* internalisation and that CD81 inhibited in MNGC formation. By blocking with a specific antibody to CD81 or recombinant CD81-EC2 protein, we found a decrease in the number and size of MNGC in infected cells. We confirmed that CD81 knock out A549 cells, in comparison to A549 wild type, had a significantly decreased MNGC formation when infected with *B. pseudomallei*. Our results in mouse macrophage J774A.1 cells were different from A549 in that both CD9 and CD81 were involved in *B. pseudomallei*-induced MNGC formation. By blocking with specific antibodies or EC2 proteins, we showed a significant decrease in MNGC formation of infected cells.

The tetraspanins CD9, CD63, and CD81 are known to be widely expressed in mammalian cells, but whereas CD9 and CD81 are predominantly expressed on the cell surface, CD63 is mainly distributed in intracellular vesicular compartments^25^. Our data are consistent with those reports confirming that CD9 and CD81 but not CD63 were expressed on the cell surface of A549 and J774A.1 cells.

Tetraspanins are known to be involved in infections caused by other intracellular pathogens, particularly viruses^4,26,27^. There is also growing evidence for roles for tetraspanins in bacterial infection; for example, CD81 has been demonstrated to be involved in *Listeria monocygenes* invasion into human epithelial cells^28^. TEMs appear to act as "adhesion platforms" for a wide range of bacteria, and that agents that disrupt TEM can prevent bacterial attachment to host cells^29–31^. *B. pseudomallei* employs several virulence factors for adhesion and invasion to host cells such as PilA and adhesion proteins; BoaA and BoaB^32^. For invasion, BopE, type III secretion system (T3SS) effectors facilitate *B. pseudomallei* entry into host cells^33^. However, our study did not find that CD9, CD63, and CD81 mediate the adherence of *B. pseudomallei* to epithelial cells and macrophages. The interplay between host cells and *B. pseudomallei* is complex and not very well understood but presumably in macrophage uptake is mainly by phagocytosis.

Interestingly, our results demonstrated that anti-CD9 MAb and CD9-EC2 but not CD63 or CD81 reagents enhanced *B. pseudomallei* internalisation into A549 cells. The data suggest that anti-CD9 MAb or CD9-EC2 may stabilise or enhance the formation of entry platforms for bacteria. The interaction of CD9 with *B. pseudomallei* components that explains its involvement with bacterial internalisation is currently unknown. Further study is required to investigate the role of CD9 as well as other tetraspanins for *B. pseudomallei* internalisation and to identify their partner proteins for the organisation of "entry platforms" for *B. pseudomallei* infection. Our data cannot suggest that overexpression of CD9 in A549 leads to a decrease in internalisation during *B. pseudomallei* infection but data from Hassuna NA et al. suggest that overexpression of CD63 in HEK 293 cells is not sufficient to promote *S.* Typhimurium binding to non-phagocytic cells^30^.

There was no effect of CD9 or CD81 reagents on J774A.1 cells internalisation which might be expected to occur via phagocytosis but we observed the effect in A549 cells, where the mechanism presumably involves the T3SS. This suggests that the tetraspanins were not affecting phagocytosis^34^ but may be affecting elements of the T3SS-mediated entry. CD81 has been shown to be necessary for internalisation of *L. monocytogenes* in which the mechanism involving phosphatidylinositol 4-kinase II^28^. CD81 might also affect actin polymerization, e.g., CD81 interfaces between the plasma membrane and the cytoskeleton by activating spleen tyrosine kinase, colocalizing ezrin-radixin-moesin proteins, and recruiting F-actin to facilitate cytoskeletal reorganization and cell signaling^35^. CD9 and CD81 have also been shown to be linked to the actin cytoskeleton via their interaction partner proteins EWI-2 and EWI-F^36^. Our data indicate that CD81 plays a significant role in MNGC formation in both epithelial cells and macrophages. In contrast, the data indicate that CD9 plays this role in macrophage cells only. The finding from our CRISPR/Cas9 knockout experiment in A549 cells has consistently confirmed the role of CD81 in *B. pseudomallei*-induced MNGC formation and CD9 in internalization. Although the CD9 and CD81 genes knockout experiment in J774A.1 cells was not successful, our data suggest that CD9 and CD81 are involved in *B. pseudomallei*-induced MNGC formation in this cell. The evidence for this belief comes from our consistent results that the antibodies and EC2 proteins of CD9 and CD81 can block *B. pseudomallei*-induced MNGC formation.

Our immunofluorescence and confocal microscopy analyses showed that CD9 and CD81 were distributed widely on the cell surface and located with *B. pseudomallei* during cell fusion. Other studies have also demonstrated the relevance of CD9 and CD81 on cell-to-cell fusion in other conditions. For example, anti-mouse CD9 and CD81 MAbs can inhibit sperm-oocyte fusion^37^. Gene knockout of CD9 or CD81 in mice could impair sperm-egg fusion while double knockout of CD9 and CD81 were completely interfile^38^. Anti-CD9 MAb can inhibit canine distemper virus-induced cell-to-cell fusion^12–15^. CD9-EC2 can inhibit monocyte fusion and reduce MNGC size in monocytes cultured under fusogenic conditions^39–41^. To our knowledge, the role of tetraspanins in MNGC formation has never been reported in *B. pseudomallei*. It is possible that these tetraspanins may not directly interact with bacterial molecules during the infection and MNGC formation processes, but they may be regulated by other host partner TEM proteins that may directly interact with *B. pseudomallei*. Other host proteins have been shown to be involved with *B. pseudomallei* infection including E-selectin (CD62E), a fusion regulatory protein (CD98), E-cadherin (CD324) integrin (LFA-1) complex, ICAM-1 (CD54), signal-regulatory protein α (CD172a), and integrin-associated protein (CD47). Blocking these proteins with specific antibodies could inhibit *B. pseudomallei*-induced MNGC formation in human leukemic monocyte lymphoma cell line^42^. We also showed the effects of antibodies to CD47, CD98, and dendritic cell-specific transmembrane protein (DC-STAMP) on *B. thailandensis-*induced fusion in J774.2 cells^16^. CD9 and CD81 that are involved in *B. pseudomallei*-induced MNGC formation may be associated with other cell surface molecules that may interact with *B. pseudomallei*. The antibodies and recombinant EC2 proteins target the EC2 regions of the tetraspanins. However, if the mechanism of action is affecting signalling in the cells, then this could be by the recruitment of intracellular signalling molecules via the C-terminal regions of the tetraspanins. Moreover, tetraspanin peptides are active on the host cell rather than on the bacteria themselves, as they have no toxicity to the bacteria, e.g., they have no anti-*S. aureus* adherence activity when added to the bacteria before adhesion to host cells^31^. Blocking of the EC2 domain of tetraspanins by antibodies may interfere with the association between tetraspanins and other partner molecules, leading to alteration of TEM organization and function^43^. This vital information warrants further investigation.

The effect of anti-tetraspanin MAbs on MNGC formation induced by *B. thailandensis* was different from those induced by *B. pseudomallei*. Specifically, anti-CD9 and anti-CD81 enhanced MNGC formation induced by *B. thailandensis* in both A549 and J774A.1 cells, whereas anti-CD9 and anti-CD81 inhibited MNGC formation induced by *B. pseudomallei*, suggesting that the roles of tetraspanins for cell–cell fusion are dependent on the microorganism. Although *B. thailandensis* is a closely-related species to *B. pseudomallei* and widely used as a model to study in melioidosis, our study suggests different effects of host tetraspanins transmembrane in the bacterial-induced cell fusion of these two species. The mechanism by which tetraspanins are involved in cell–cell fusion after *Burkholderia* infection is not well understood. Our data and data from other studies suggest that host factors and probably bacterial factors seem to be involved in the cell-to-cell spread process. The MNGC formation induced by *B. pseudomallei* or *B. thailandensis* requires the type VI secretion system (T6SS-5), which is regulated by BsaN, VirAG, and BprC^18,44^. This mechanism could lead to cell death by activating autophagy and triggering the cGAS-STING pathway via micronuclear formation^20^. T6SS-5 plays an essential role in MNGC formation by which the C-terminal of VgrG5 processes fusogenic activity^18,45^. It is likely that the different amino acid sequences and conformation of these factors^45,46^ might contribute to the interaction with associated tetraspanins. It will be of interest to determine the effect of CD9 or CD81 on MNGC formation during infection with *B. pseudomallei* T6SS-5 and VgrG5 mutants in future studies.

It is not clear why MAb treatments were more efficient in inhibition of MNGC formation and subsequent reduction of MNGC size than recombinant EC2 protein treatments. A possible explanation is that the MAbs may be more specific to the disruption of tetraspanin function compared to the recombinant EC2 proteins.

Tetraspanins have been reported as a novel treatment for parasitic and bacterial infections. Synthetic peptides of tetraspanin, CD9 prevent *Staphylococccus aureus* skin and wound infections to cultured keratinocytes and human skin infection model^31^. Our finding suggests that targeting these tetraspanins using specific antibodies or recombinant EC2 proteins could inhibit *B. pseudomallei*-induced MNGC, thus limited bacterial spreading between different cell types and that CD9 and CD81 may be good vaccine targets. Antibodies or recombinant EC2 proteins may be useful for therapeutic purposes. However, this requires further evaluation in other cell types and testing in animal models of melioidosis.

Host and bacterial interplay are complicated, but our study provides insights into the understanding of *B. pseudomallei*-induced cell-to-cell fusion of human epithelial and mouse macrophage cells by which tetraspanins are associated with the process by their distribution on the cell surface. Our study in epithelial cells and macrophages revealed that CD9 and CD81 are involved in *B. pseudomallei*-induced MNGC formation. Antibodies specific to tetraspanins and EC2 proteins could be a potential therapeutic agent to prevent the spread of *B. pseudomallei* to uninfected cells.

## Methods

### Bacterial strain, cell lines, and growth conditions

*B. pseudomallei* strain K96243, a clinical isolate, and *B. thailandensis* strain E264, an environmental isolate from Thailand, were used in this study. The bacteria were grown on Columbia agar (Oxoid, UK) and incubated at 37 °C overnight. Human lung epithelial cell A549 (CCL-185, American Type Culture Collection, MD, USA) was cultured in RPMI 1640 (Gibco BRL, Grand Island, NY, USA) supplemented with 10% heat-inactivated fetal bovine serum (FBS) (HyClone, USA). Mouse macrophage cell line J774A.1 was cultured in DMEM (Gibco BRL). All cell cultures were performed at 37 °C in a humidified 5% CO_2_ incubator. All experiments involving *B. pseudomallei* were performed in a biosafety level 3 laboratory.

### Antibodies against tetraspanins and recombinant GST fusion proteins

Three monoclonal antibodies (MAbs) that recognised human tetraspanins CD9 (clone 602.29), CD63 (clone H5C6), CD81 (clone 1D6, Bio-Rad, USA), and IgG1 isotype control (clone JC1) were used to treat A549 cells. MAbs that recognised mouse tetraspanins, including MAbs specific to CD9 (clone MF1, Bio-Rad), CD63 (clone NVG-2, BioLegend Inc., San Diego, CA, USA), CD81 (clone Eat2, Bio-Rad), and rat IgG2b, rat IgG2a, and hamster IgG1 isotype controls were used to treat J774A.1 cells. The production of recombinant glutathione S-transferase (GST) fusion proteins with CD9, CD63, and CD81 tetraspanin EC2 extracellular domains were previously described^30^.

### Examination of tetraspanin expression

Cell surface expression of tetraspanins on A549 and J774A.1 cells was assessed using flow cytometry. The experiment was performed as previously described^30^. The cells were cultured in the medium for 2 days. To remove cells from culture flasks, A549 cells were trypsinised with 0.25% of trypsin–EDTA (Gibco BRL), while J774A.1 cells were dislodged by cell scraper. The cells at a concentration of 1.0 × 10^5^ cells were incubated with 5 µg/ml of each primary Mab against tetraspanins, or isotype control at 4 °C for 1 h. The cells were then washed with PBS followed by incubating with 5 µg/ml secondary antibodies (A549: Alexa Fluor 488 conjugated goat anti-mouse antibody (Invitrogen, Waltham, MA, USA) or FITC-conjugated anti-rat IgG; J774A.1: anti-hamster IgG (Bio-Rad, USA) at 4 °C for 30 min. The cells were centrifuged at 1,000 rpm at 4 °C for 5 min, washed with PBS, and analysed using a FACSCalibur flow cytometer (Becton Dickinson, Franklin Lakes, NJ, USA). The data was analysed using a Flowjo software version 10.1 (FlowJo, LLC). Two independent experiments were performed for each experiment.

### Adhesion assay

A549 and J774A.1 cells were seeded at 1 × 10^4^ cells in 100 µl per well into a 96-well plate and incubated at 37 °C for overnight. The cells were pretreated with 20 µg/ml anti-tetraspanin MAbs or recombinant EC2 proteins at 37 °C for 1 h, then washed with PBS and infected with *B. pseudomallei* K96243 at a multiplicity of infections per cell (MOI) at 100 for A549 and 30 for J774A.1. The plates were incubated at 37 °C with 5% CO_2_ for 1 h. Non-adherent bacteria were detached by gentle washes with PBS for 5 times^47^, and the cells were lysed with 0.1% Triton X-100 (Sigma-Aldrich) for 5 min. The bacterial suspension was serially diluted with PBS, spread on Columbia agar, and incubated at 37 °C for 24 h for colony count. The number of adhered bacteria was enumerated as colony-forming units (CFU). The percentage of adhesion reflected the number of adherent bacteria at 1 h post-infection × 100/ the number of CFU added. The percentage of adhesion was calculated from three independent experiments; each was conducted in triplicate^47^.

In another experiment, J774A.1 cells were pretreated with 2 µg/ml of cytochalasin D (Sigma-Aldrich) for 2 h to block phagocytosis^17^. The adhesion assay was then performed as described above.

### Internalization assay

A549 and J774A.1 cells were seeded and pretreated with anti-tetraspanin MAbs or recombinant EC2 proteins, as described above. The cells were then washed with PBS and infected with *B. pseudomallei* at MOI of 100 for A549 and 30 for J774A.1 for 2 h to allow bacterial internalisation. The cells were washed with PBS before adding a fresh culture medium containing 250 mg/ml of kanamycin (Gibco BRL). The infected cells were further incubated for 2 h to kill residual extracellular bacteria. The infected cells were then washed and lysed with 0.1% Triton X-100. The intracellular bacteria were colony counted. The percentage of internalization reflected the number of intracellular bacteria at 4 h post-infection × 100/ the number of bacteria added. The percentage of internalization and MNGC formation was calculated from three independent experiments; each was conducted in triplicate^47^.

### MNGC formation assay

The determination of MNGC formation was performed with *B. thailandensis* E264 and *B. pseudomallei* K96243, as described by Kespichayawattana W et al.^19^. A549 and J774A.1 cells were seeded in a 96-well plate and pretreated with anti-tetraspanin MAbs or recombinant EC2 proteins, as described above. The cells were washed and infected with bacteria at MOI of 100 for A549 cells and 30 for J774A.1 cells for 2 h. The extracellular bacteria were then removed, and the cells were further incubated with a fresh culture medium containing 250 mg/ml of kanamycin. MNGC formation was quantified after 12 h of incubation. The cells were washed with PBS, fixed with 4% paraformaldehyde in PBS for 30 min, washed again with PBS, and stained with Giemsa solution (Merck KGaA, Darmstadt, Germany) for 5 min. The cells were rewashed with water and air-dried at RT. The stained cells were imaged with an Operetta High Content analysis system (PerkinElmer) and analyzed using ImageJ software version 1.52n (https://rsb.info.nih.gov/ij/). The cells with 3 nuclei or more were considered to be MNGC. The percentage of MNGC was calculated from many nuclei in MNGC × 100/total number of nuclei counted. MNGC size was calculated from the number of nuclei in MNGC/number of MNGC^40^. The percentage of MNGC formation was calculated from three independent experiments, each of which was conducted in triplicate.

### Immunostaining and confocal microscopy

Immunostaining was performed with CD81 for A549 cells and CD9 for J774A.1 cell, as previously described^48^. A549 or J774A.1cells were seeded at 7 × 10^5^ cells/well on a sterile glass coverslip in a 6-well cell culture plate and incubated overnight at 37 °C with 5% CO_2_. The monolayer of cells was infected with *B. pseudomallei* at MOI of 25:1 and 10:1 for two h washed with PBS, and further incubating with a medium containing 250 µg/ml of kanamycin for 6 h. The cells were washed with PBS and fixed with 4% paraformaldehyde in PBS for 30 min. The cells were permeabilised with 0.5% triton X-100 for 30 min. The permeabilised cells were then incubated at 37 °C for 1 h with a mixture of (i) an Alexa Fluor 488 conjugated mouse MAb 4B11 specific to *B. pseudomallei* capsular polysaccharide^49^ at dilution of 1:200, (ii) 20 µg/ml of an Alexa Fluor 555 conjugated mouse anti-human CD81 or Alexa Fluor 555 conjugated rat anti-mouse CD9 and (iii) Hoechst 33,258 (Invitrogen) at dilution of 1:1000. MAbs 4B11 was conjugated with an Alexa Fluor 488 (Invitrogen) and mouse anti-human CD81 or a rat anti-mouse CD9 antibodies or isotype-matched controls were conjugated with an Alexa Fluor 555 according to the manufacturer's instruction (Invitrogen). After three times washing with PBS, coverslips were mounted on a glass slide with 8 µl of ProLong Gold antifade reagent (Invitrogen). The cells were imaged using a laser scanning confocal microscope (Carl Zeiss LSM 700; Jena, Germany) by Z-stack mode with an oil-immersion 100 × objective lens and analysed with a ZEN 2010 version 6.0 software.

### Generation of tetraspanins knock out cell lines

*CD9* and *CD81* genes knock out were performed with A549 and J774A.1 cells using a CRISPR/Cas9 system^50^. Single guide RNA (sgRNA) sequences targeting both mouse and human CD9 and CD81 were designed with appropriate overhangs: sgRNA1 (Human CD9-Fw 5′-CACCTTAGGGCGCGCGACCCGCTG-3′ and Human CD9-Rw 5′-AAACCAGCGGGTCGCGCGCCCTAA-3′); sgRNA2 (Human CD81-Fw 5′-CACCCCTCCACTCCCATGGCGGCG-3′ and Human CD81-Rw 5′-AAACCGCCGCCATGGGAGTGGAGG-3′); sgRNA3 (Mouse CD9-Fw 5′-CACCCTGAGACACGCTGCGGCTAG-3′ and Mouse CD9-Rw 5′-AAACCTAGCCGCAGCGTGTCTCAG-3′); sgRNA4 (Mouse CD81-Fw 5′- CACCTGGGGACAGGGCGCACCCCA-3′ and Mouse CD81-Rw 5′-AAACTGGGGTGCGCCCTGTCCCCA-3′). sgRNA sequences targeting both mouse and human CD9 and CD81 were cloned into the Bbs1 site of the pSpCas9(BB)-2A-Puro V2.0 (PX459V2.0; Addgene, Cambridge, MA, USA) by using single-step digestion-ligation and a standard cloning method^51^. The inserted gRNA sequences were verified by direct sequencing. A549 or J774A.1 cells were transfected for 24 h with individual plasmids expressing Cas9 and the corresponding sgRNA using lipofectamine 3,000 reagent (Invitrogen, Waltham, MA, USA) according to the manufacturer's instruction. After transfection, the cells with plasmids were selected by incubating in culture media containing puromycin (2 μg/mL) for 48 h. Both CD9 and CD81 negative cells were sorted into a 5 ml polystyrene round-bottom tube (Corning, New York, USA) using a BD FACSAria III (Becton Dickinson, Franklin Lakes, NJ, USA). Single-cell clones were isolated by limiting dilution in a 96 well plate and expanded for gene knockout validation.

### Validation of tetraspanins knockout cell lines

Genomic DNA was isolated from individual single-cell clones and non-edited control cells using the Gentra Puregene Blood Kit (Qiagen, Hilden, Germany). PCR amplicons spanning the Cas9-sgRNA cleavage site was amplified in a 25 µl total reaction volume consisting of 1 µl of 50 ng DNA template, 12.5 µl of KAPA2G Fast Hotstart PCR kit (Kapa Biosystems, Inc., Wilmington, MA, USA), 0.5 µl of each 10 µm forward and reverse primers with the following forward and reverse primers: CD9: CD9F 5′-GCGAGGCCTCCACGTAAGTC-3′ and CD9R 5′-AACTTAGCCTGGGCCGAACG-3′; or CD81: CD81F 5′- ATAAGTACTGCGGAGCGAGG-3′ and CD81R 5′-CTCCTGGAGCCCACAGGTGG-3′. The PCR conditions were as follows: initial denaturation at 95 °C for 3 min; 34 cycles of denaturation at 95 °C for 15 s, annealing at 60 °C for 10 s, and extension at 68 °C for 20 s; followed by a final extension at 68 °C for 5 min. The PCR amplicons were run on a 2% agarose gel. Each PCR fragments were purified and subjected for direct sequencing using the forward and reverse PCR.

### Western blot analysis

Knock out cells were tested for the presence of CD9 and CD81 at the protein level using 15% SDS-PAGE and Western blotting. A549 cells were lysed with 100 µl lysis buffer (25 mM Tris–HCl pH 7.4 containing 150 mM NaCl, 1% sodium deoxycholate, 1% TritonX-100, 0.1% SDS and 1 mM EDTA and 5% glycerol) and 1 µl protease inhibitor (Halt protease and phosphatase inhibitor cocktail (100X) (Thermo Fisher, Waltham, MA, USA) at 4 °C for 30 min. The lysed cells were centrifuged at 15,000 × *g* at 4 °C for 15 min. The protein concentration of the supernatant was quantified using a BCA kit (Thermo Fisher, Waltham, MA, USA). Cell lysates were diluted with sample buffer and heated at 100 °C for 5 min. Thirty µg of protein was run on 15% SDS gels, transferred to a nitrocellulose membrane and probed with monoclonal anti-CD9 antibody (clone 602.29) or anti-CD81 antibody (clone 1D6, Bio-Rad, USA) at dilution of 1:1000. Rabbit anti-mouse IgG/HRP (Dako Cytomation, Denmark) at dilution of 1:2000 was used as secondary antibody. BM Chemiluminescence Blotting Substrate (Roche, Germany) was added on the membrane before exposure to an X-ray film.

### Statistical analysis

Statistical analysis was performed using GraphPad Prism software version 6.0 (GraphPad Software Inc, La Jolla, CA). The data are presented as individual points and means ± standard deviation. The data were tested for the difference using one-way ANOVA for comparison among three or more groups and *t* tests for comparison between two groups. A statistically significant difference was considered at *P* value < 0.05.

## Supplementary information

Supplementary Figures
