# Supplementary material for: Tetraspanins are involved in Burkholderia pseudomallei-induced cell-to-cell fusion of phagocytic and non-phagocytic cells
Source: Sci Rep. 2020 Oct 21;10:17972. doi: 10.1038/s41598-020-74737-y (PMC7577983; doi:10.1038/s41598-020-74737-y)

**Tetraspanins are involved in *Burkholderia pseudomallei*-induced cell-to-cell fusion of phagocytic and non-phagocytic cells**

Tanes Sangsri^1^, Natnaree Saiprom^1^, Alisa Tubsuwan^2^, Peter Monk^3^, Lynda J. Partridge^4^, Narisara Chantratita^1, 5*^

^1^Department of Microbiology and Immunology, Faculty of Tropical Medicine, Mahidol University, Bangkok 10400, Thailand. E-mail: tanes.sn@gmail.com

^1^Department of Microbiology and Immunology, Faculty of Tropical Medicine, Mahidol University, Bangkok 10400, Thailand. E-mail: ningkhub@hotmail.com

^2^ Institute of Molecular Biosciences, Mahidol University, Nakhon Pathom 73170, Thailand. Email: alisa.tub@mahidol.ac.th

^3^Department of Infection, Immunity and Cardiovascular Disease, School of Medicine, University of Sheffield, Beech Hill Road, Sheffield, S10 2RX, UK.
**E-mail**: p.monk@sheffield.ac.uk

^4^Department of Molecular Biology and Biotechnology, University of Sheffield, Western Bank, Sheffield, S10 2TN, UK. E-mail: l.partridge@sheffield.ac.uk

^5^Mahidol-Oxford Tropical Medicine Research Unit, Faculty of Tropical Medicine, Mahidol University, Bangkok 10400, Thailand. E-mail: narisara@tropmedres.ac

***Corresponding author:** Narisara Chantratita, Department of Microbiology and Immunology, Faculty of Tropical Medicine, Mahidol University, 420/6 Rajvithi Road, Bangkok 10400, Thailand. Tel: (+66) 819099772, E-mail: narisara@tropmedres.ac

**Supplementary materials**

**Supplementary Figure S1.** Surface expression of CD9, CD63, and CD81 on uninfected A549 and J774A.1 cells. The cells were cultured at 37°C with 5% CO_2_ atmosphere for 48 h. The analysis was performed by flow cytometry on A549 cells (A) and J774A.1 cells (B). Values are the median fluorescence intensities (MFI) of 10,000 gated cells ± standard deviation from two independent experiments. The graphs were created using GraphPad Prism software version 6.0 (GraphPad Software Inc, La Jolla, CA).

**
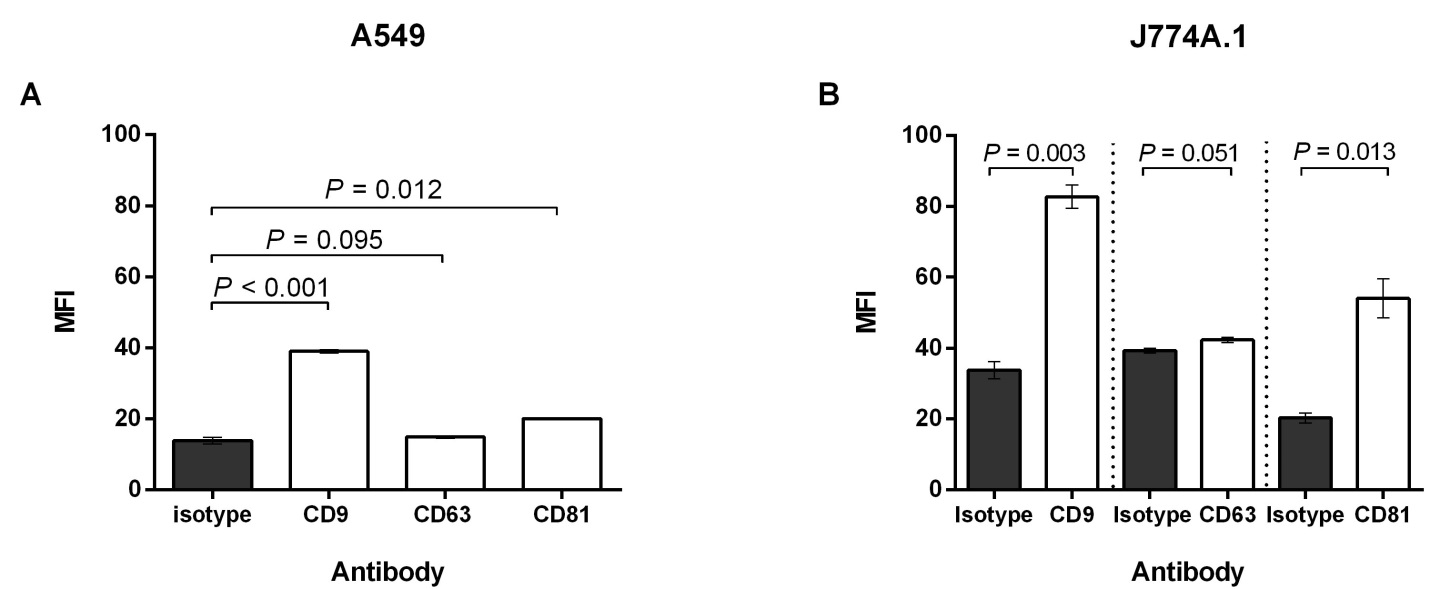
**

**Supplementary Figure S2.** Analysis of tetraspanin expression of A549 and J774A.1 cells. The median fluorescence intensity (MFI) was calculated from the MFI of uninfected cells (U) and infected cells (I) at three different time points (1, 4, and 12 h). A-C show the MFI of tetraspanin expression on A549 cells at 1, 4 and 12 h, respectively. D-F show the MFI of tetraspanin expression in J774A.1 cells at 1, 4, and 12 h, respectively. Data represent the mean ± standard deviation from two independent experiments. The graphs were created using GraphPad Prism software version 6.0 (GraphPad Software Inc, La Jolla, CA).

**
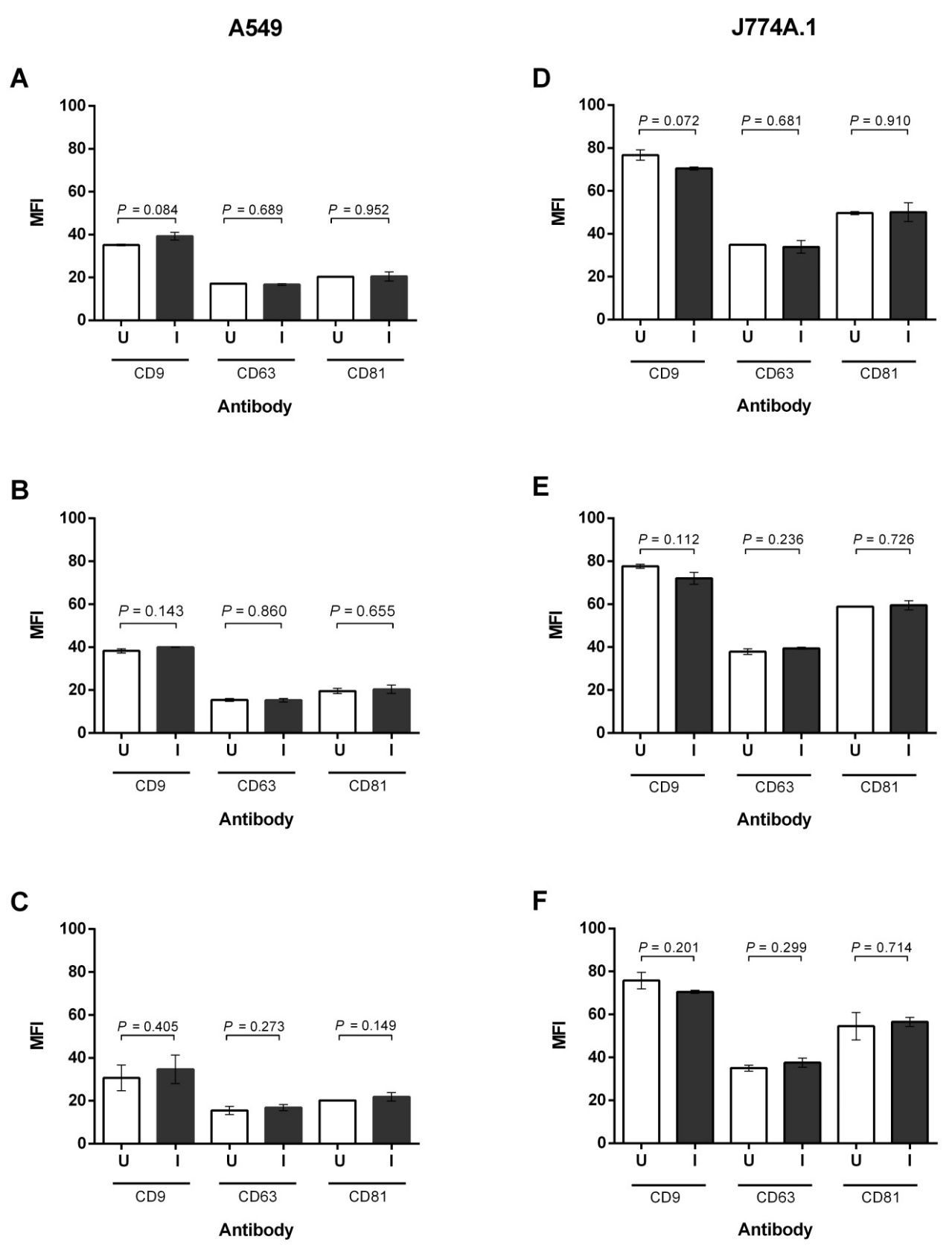
**

**Supplementary Figure S3.** Effect of anti-tetraspanin MAbs and recombinant EC2 proteins pretreatment on *B. pseudomallei* K96243 adhesion to A549 and J774A.1 cells. Adhesion of *B. pseudomallei* K96243 to A549 cells (A and B) and J774A.1 (C and D) were performed at MOI of 100 and 30, respectively. A and C show the effect of anti-tetraspanin MAbs. B and D show the effect of recombinant EC2 protein pretreatment. Data represent individual scatter plots and the mean ± standard deviation from three independent experiments; each experiment was performed in triplicate. Dashed lines represent *P* values of ANOVA test, and solid lines represent *P* value of *t* tests. The graphs were created using GraphPad Prism software version 6.0 (GraphPad Software Inc, La Jolla, CA).


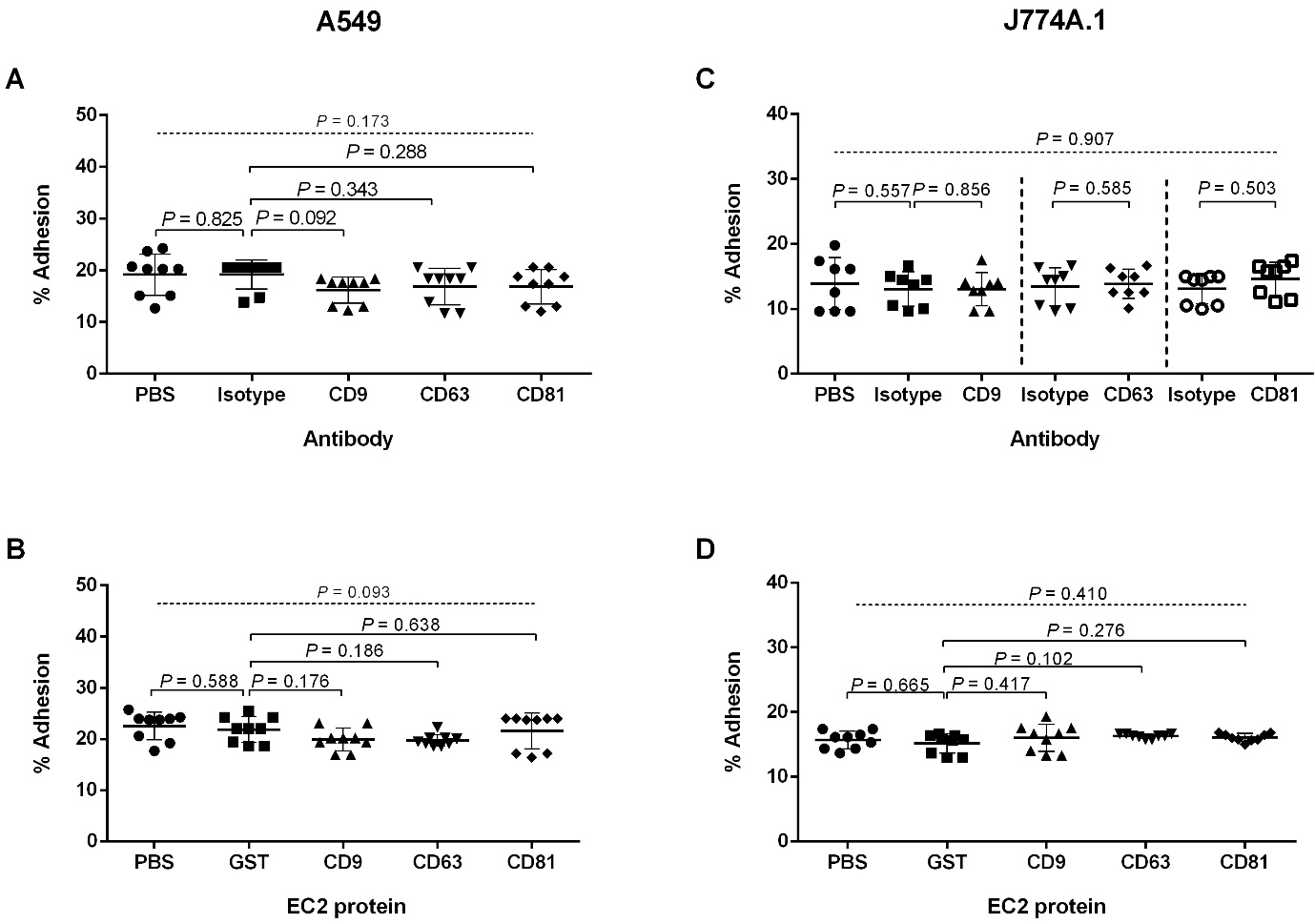


**Supplementary Figure S4.** Effect of anti-tetraspanin MAbs pretreatment on *B. pseudomallei* K96243 adhesion after blocking phagocytosis in J774A.1 cells. Cells were pretreated with 2 µg/ml of cytochalasin D for 2 h to block phagocytosis. Adhesion of *B. pseudomallei* K96243 was performed at MOI of 30. Data represent individual scatter plots and the mean ± standard deviation from three independent experiments; each experiment was performed in triplicate. Dashed lines represent *P* values of ANOVA test, and solid lines represent *P* value of *t* tests. The graphs were created using GraphPad Prism software version 6.0 (GraphPad Software Inc, La Jolla, CA).


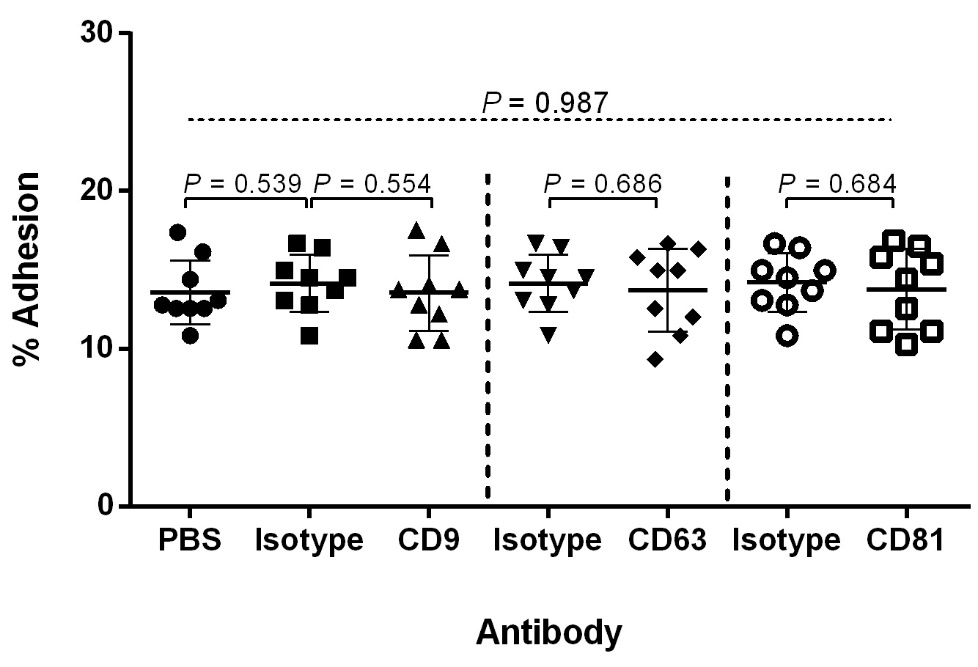


**Supplementary Figure S5.** Effect of different concentrations of CD9-EC2 protein on *B. pseudomallei*-induced MNGC formation in J774A.1 cells compared with GST control (A). Effect of CD9-EC2 or CD63-EC2 or CD81-EC2 alone and a combination of CD9-EC2 and CD81-EC2 proteins at 20 µg/ml of each on *B. pseudomallei*-induced MNGC formation to J774A.1 cells compared with GST control (B). Data represent individual scatter plots and the mean ± standard deviation from two independent experiments; each experiment was performed in triplicate. The graphs were created using GraphPad Prism software version 6.0 (GraphPad Software Inc, La Jolla, CA).


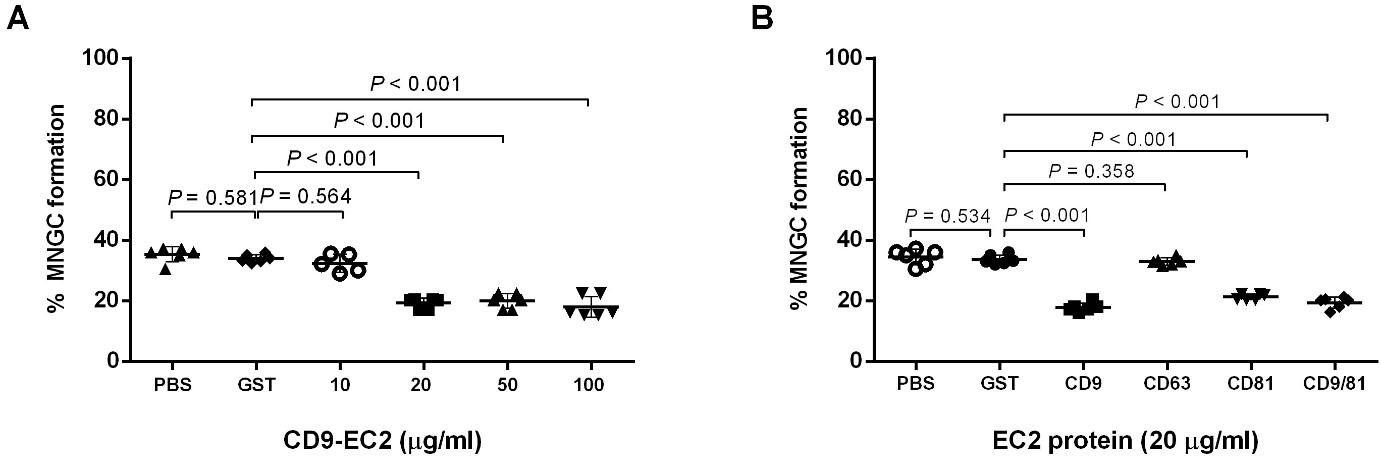


**Supplementary Figure S6.** Effect of anti-tetraspanin MAbs treatment at 5 h after infection on *B*. *pseudomallei* K96243-induced MNGC formation on in A549 and J774A.1 cells. A549 and J774A.1 cells were infected with *B. pseudomallei* at MOI of 100 and 30, respectively, and anti-tetraspanin MAbs were added at 5 h post-infection. A and B show the effect of anti-tetraspanin MAbs on *B*. *pseudomallei*-induced MNGC formation and average MNGC size in A549 cells. C and D show the effect of anti-tetraspanin MAbs on *B*. *pseudomallei*-induced MNGC formation and average MNGC size in J774A.1 cells. Data represent individual scatter plots and the mean ± standard deviation from three independent experiments; each experiment was performed in triplicate. Dashed lines represent *P* values of ANOVA test, and solid lines represent *P* value of *t* tests. The graphs were created using GraphPad Prism software version 6.0 (GraphPad Software Inc, La Jolla, CA).


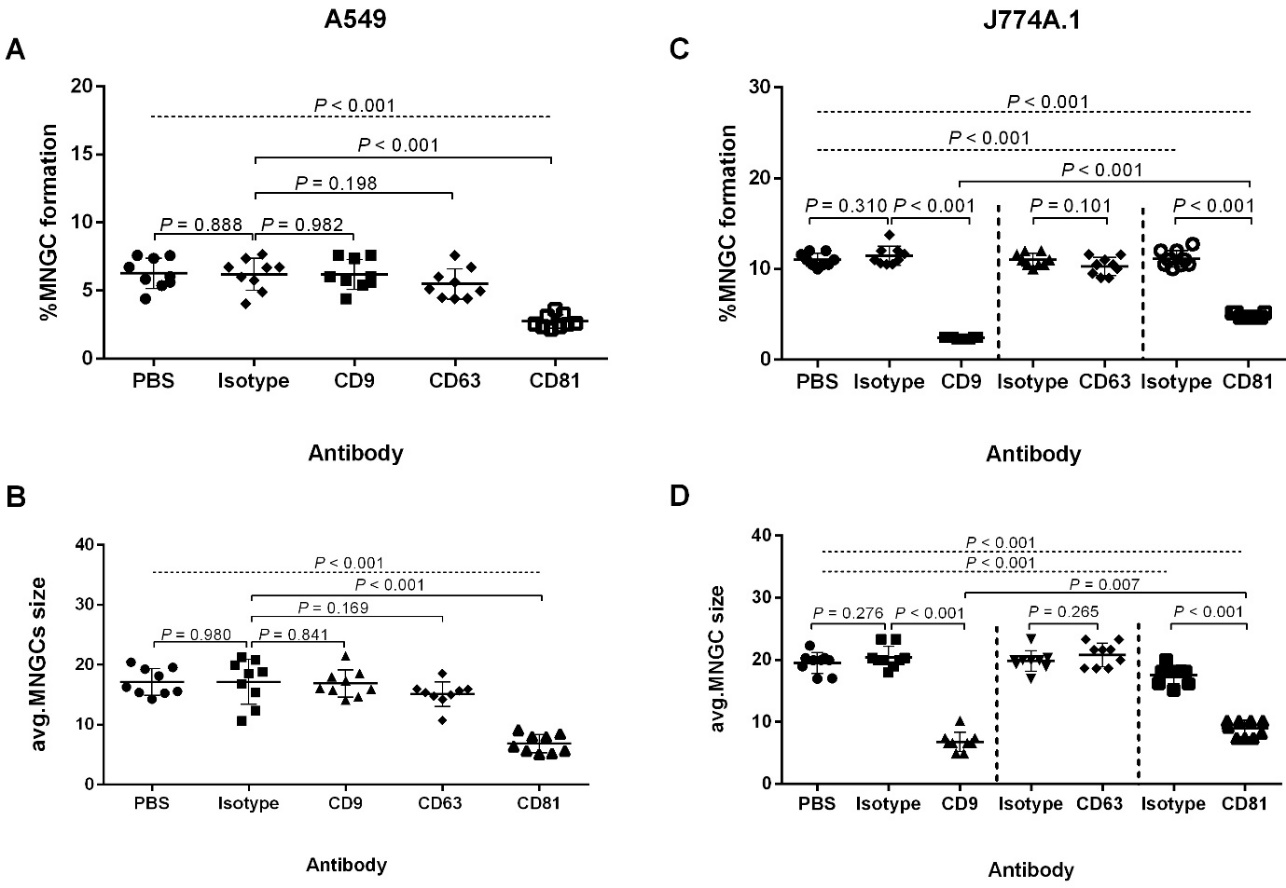


**Supplementary Figure S7.** Giemsa-stained A549 and J774A.1 cells following *B. pseudomallei* infection. Cells were pretreated with anti-tetraspanin MAbs or isotype controls before infection. Cells were fixed, stained, and observed for MNGC formation at 12 h post-infection. Arrow indicates the intracellular bacteria in the cytosol. The images were combined using BioRender.com (https://app.biorender.com/).


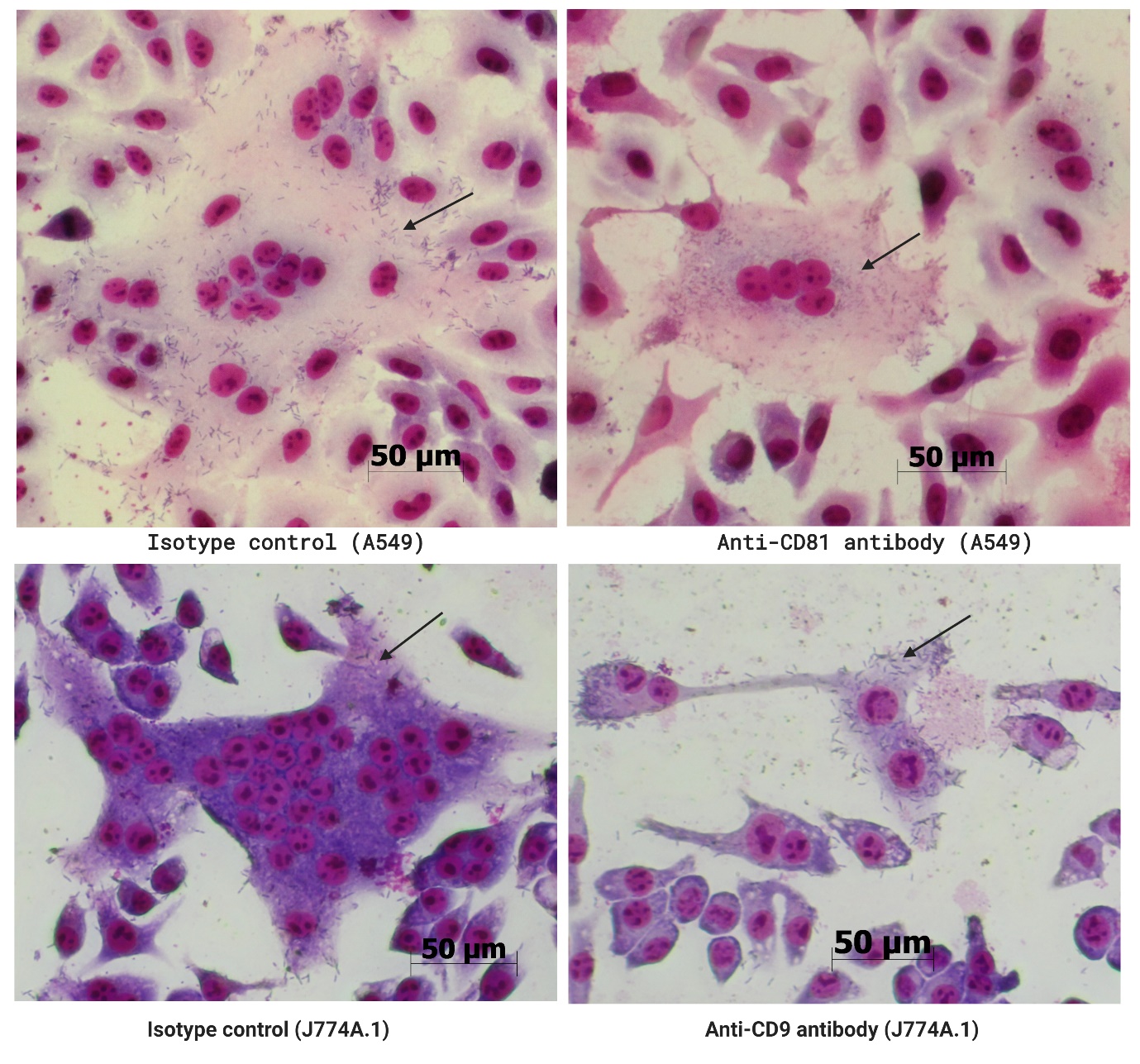


**Supplementary Figure S8.** Analysis of tetraspanin expression in J774A.1 cells by immunofluorescence microscopy. Cells were cultured on a glass coverslip, fixed and stained with anti-mouse CD9 and anti-mouse CD81 antibodies or appropriate isotype-matched controls at 20 μg/ml followed by FITC-conjugated anti-rat IgG (for CD9) or anti-hamster IgG (for CD81). Nuclei were stained with Hoechst 33258. Images were visualised using a confocal microscope with 20× objective lens. The images were combined using BioRender.com (https://app.biorender.com/).


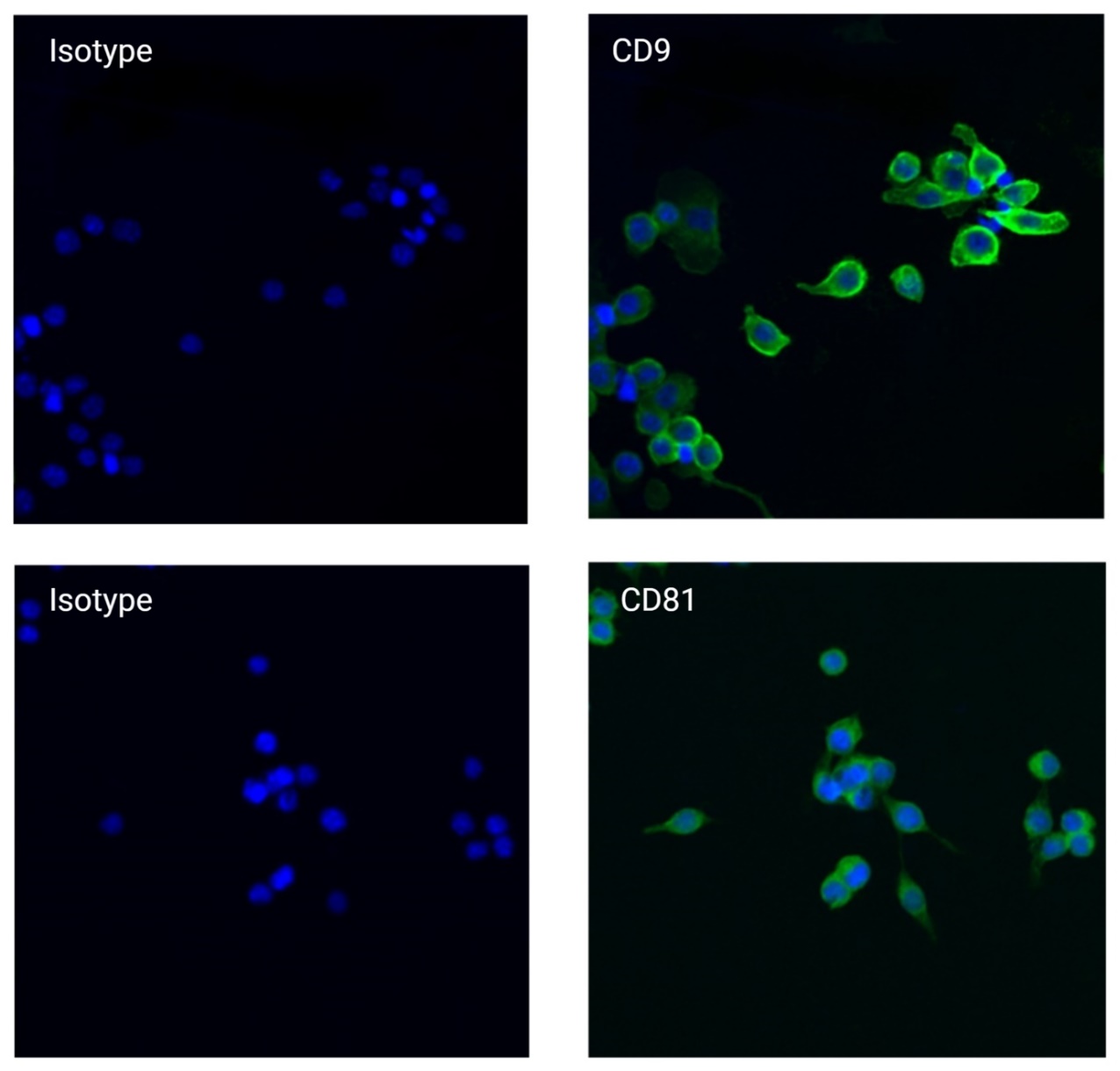


**Supplementary Figure S9.** Original gel image of PCR products of *CD9* in A549-CD9 wild type, A549-CD9 knock out mutant, *CD81* in A549-CD81 wild type and A549-CD81 knock out mutant. DNA cleavage by CRISPR/cas9 was detected in amplified products of both mutants. The images were combined using BioRender.com (https://app.biorender.com/).


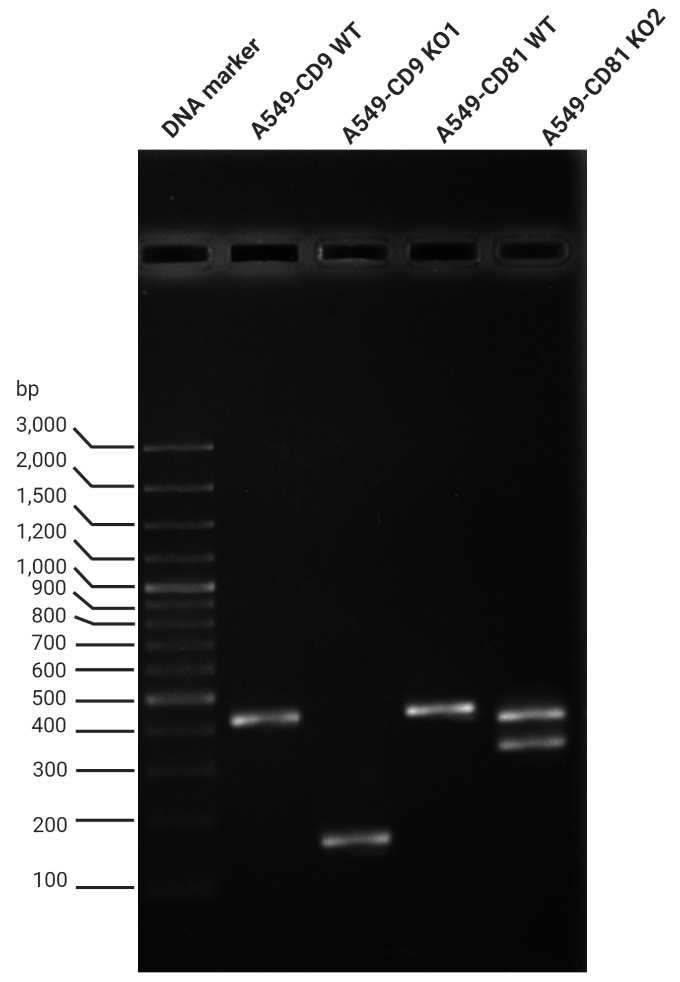


**Supplementary Figure S10. Western blot analysis of CD9 and CD81 knockouts in A549 cells**. Thirty micrograms of cell lysate were run on 15% SDS gels, transferred to a nitrocellulose membrane and probed with anti-CD9 antibody (clone 602.29) or anti-CD81 antibody (clone 1D6, Bio-Rad, USA) at a dilution of 1:1,000. Rabbit anti-mouse IgG/HRP (Dako Cytomation, Denmark) at dilution of 1:2,000 was used as a secondary antibody and BM Chemiluminescence Blotting Substrate (Roche, Germany) was used as substrate reagent. The reaction was observed on an X-ray film. Lane M, protein marker; lane 1, A549-CD9 wild type; lane 2, A549-CD9KO1; lane 3, A549-CD81 wild type; and lane 4, A549-CD81KO2. The images were combined using BioRender.com (https://app.biorender.com/).


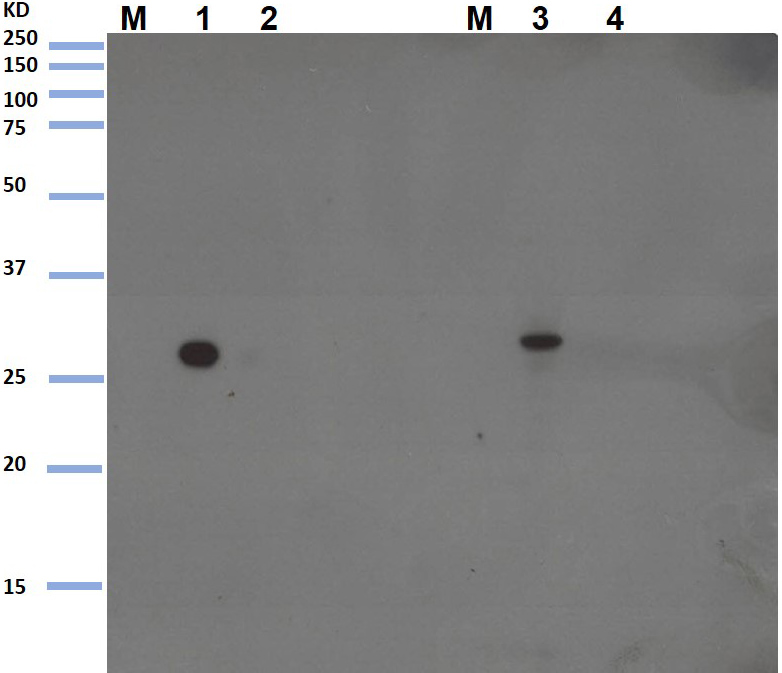

Supplement: Supplementary file 1 — Supplementary Figures [file 41598_2020_74737_MOESM1_ESM.docx]
